# O-GlcNAcylation of SIX1 enhances its stability and promotes Hepatocellular Carcinoma Proliferation

**DOI:** 10.7150/thno.45161

**Published:** 2020-08-02

**Authors:** Yi Chu, Mingzuo Jiang, Nan Wu, Bing Xu, Wenjiao Li, Haiming Liu, Song Su, Yanting Shi, Hao Liu, Xiaoliang Gao, Xin Fu, Di Chen, Xiaowei Li, Weijie Wang, Jie Liang, Yongzhan Nie, Daiming Fan

**Affiliations:** 1 State key Laboratory of Cancer Biology and National Clinical Research Center for Digestive Diseases, Xijing Hospital of Digestive Diseases, Fourth Military Medical University, Xi'an, 710032, China.; 2 Lab of Tissue Engineering, Faculty of Life Science, Northwest University, Xi'an, 710032, China.; 3 Department of Gastroenterology, Sixth Medical Center of PLA General Hospital, Beijing, 100048, China.

**Keywords:** SIX1, O-GlcNAcylation, hepatocellular carcinoma, Warburg effect, ubiquitination

## Abstract

It is universally accepted that aberrant metabolism facilitates tumor growth. However, how cancer cells coordinate glucose metabolism and tumor proliferation is largely unknown. Sine oculis homeobox homolog 1 (SIX1) is a transcription factor that belongs to the SIX family and is believed to play an important role in the regulation of the Warburg effect in tumors. However, whether the role of SIX1 and the molecular mechanisms that regulate its activity are similar in hepatocellular carcinoma (HCC) still needs further investigation.

**Methods:** Western blotting was performed to determine the levels of SIX1 and O-linked β-N-acetylglucosaminylation (O-GlcNAcylation) in HCC tissues. Cell Counting Kit 8 (CCK8), colony formation and mouse tumor model assays were used to establish the role of SIX1 and O-GlcNAcylation in HCC processes. Mass spectrometry, immunoprecipitation and site-directed mutagenesis were performed to confirm the O-GlcNAcylation of SIX1.

**Results:** Here, we demonstrated that SIX1, the key transcription factor regulating the Warburg effect in cancer, promotes HCC growth *in vitro* and *in vivo*. Furthermore, we revealed that SIX1 could also enhance the levels of a posttranslational modification called O-GlcNAcylation. Importantly, we found that SIX1 was also highly modified by O-GlcNAcylation and that O-GlcNAcylation inhibited the ubiquitination degradation of SIX1. In addition, site-directed mutagenesis at position 276 (T276A) decreased the O-GlcNAcylation level and reversed the protumor effect of SIX1.

**Conclusions:** We conclude that O-GlcNAcylation of SIX1 enhances its stability and promotes HCC proliferation. Our findings illustrate a novel feedback loop of SIX1 and O-GlcNAcylation and show that O-GlcNAcylation of SIX1 is an important way to coordinate glucose metabolism and tumor progression.

## Introduction

Reprogramming of metabolism plays an important role in tumor progression. Among the aberrant metabolic pathways of liver cancer, the Warburg effect has been widely recognized as an important feature of tumor metabolism 1. Under this effect, tumor cells consume a large amount of glucose but cannot produce sufficient energy, which promotes glucose uptake and lactic acid production, and creates a microenvironment suitable for the survival of tumor cells. The rapid increase in glucose and glutamine uptake by tumor cells 2 can alter multiple metabolic and signaling pathways in cancer cells including, for example, the hexosamine biosynthetic pathway (HBP) 3. As a crucial pathway of the cellular metabolism, HBP is at the nexus of cancer cell metabolism.

The subsequent glycosylation whose substrates are provided by the hexosamine biosynthetic pathway called O-GlcNAcylation also increases in the process of tumorigenesis 4.O-GlcNAcylation is a posttranslational modification that modifies proteins on their serine/threonine residues and is associated with the expression, location and function of O-GlcNAcylated proteins 5. This modification adds uridine diphosphate N-acetylglucosamine (UDP-GlcNAc) on a wide range of proteins solely through the activity of O-GlcNAc transferase (OGT) and could be reversed by glycoside hydrolase O-GlcNAcase (OGA) 6. O-GlcNAcylation occurs on numerous classes of proteins and acts as a mediator that coordinates cellular processes such as signal transduction, transcription, translation, and protein degradation 7. An enhanced O-GlcNAcylation level has been reported in various kinds of tumors 8-10 and OGT also has been described to be increased in myriad cancers such as breast, prostate, and ovarian cancer 11-13. However, how the glucose metabolism regulates O-GlcNAcylation and what are the feedback effects of aberrant O-GlcNAcylation on glucose metabolism in cancer still need further investigation.

SIX1 is a transcription factor that belongs to the SIX family and is believed to play an important role in the regulation of the Warburg effect in tumors. SIX1 could interact with the histone acetyltransferase HBO1 and AIB1 to activate the expression of a series of glycolytic genes 14, which can stimulate the process of cell glycolysis and ultimately promotes cancer progression. SIX1 has been found to be overexpressed in various kinds of caner 15-17 and is known to be involved in the tumor progression in both proliferation and metastasis 18-21. Since HBP is dependent on the availability of glucose, whether SIX1, a regulator of Warburg effect, could also impact on HBP and subsequent O-GlcNAcylation in HCC still remain unclear and investigating the potential relationship between SIX1, HBP and O-GlcNAcylation will improve our understanding of the function of SIX1 in HCC. Moreover, SIX1 has been found to be regulated by posttranslational modifications such as phosphorylation and ubiquitin - mediated proteolysis 22, 23. However, as O-GlcNAcylation is a post translational modification with an emerging role in the regulation of metabolic factors, the relationship between O-GlcNAcylation and SIX1 has not yet been explored, which may link O-GlcNAcylation to the Warburg effect and other key molecular pathways that occur during cancer.

In the present study, we investigated the potential functions of SIX1 and O-GlcNAcylation in HCC progression and found a correlation between the aberrant glucose metabolism and O-GlcNAcylation. We found that SIX1 and O-GlcNAcylation levels were upregulated both in the clinical samples and HCC cell lines. We also found that SIX1 could increase O-GlcNAcylation levels and that hyper O-GlcNAcylation could enhance SIX1 stability by inhibiting SIX1 protein ubiquitination progression. Furthermore, mutating the threonine at position 276 (T276A) decreased the O-GlcNAcylation of SIX1 and enhanced its degradation. Our data provide the initial evidence that O-GlcNAcylation of SIX1 promotes HCC progression, suggesting that inhibition of OGT and SIX1 could be a potential therapy for HCC treatment.

## Methods

### Patients and tissue samples

The current study was conducted under the supervision of the Xijing Hospital's Protection of Human Subjects Committee of the Fourth Military Medical University (Xi'an, China). Fifty paired HCC primary and adjacent normal samples were obtained from patients who underwent surgery at the Xijing Hospital. Written informed consent was obtained from all patients and relatives who donated tissue samples.

### Cell lines and cell culturing

The cell lines THLE-3, MHCC97H, HepG2, SMMC7721, Huh7, BEL7402, and BEL7404 were stored in our laboratory. All human cell lines were authenticated using STR profiling within the last three years and were cultured in Dulbecco's modified Eagle's medium (DMEM; Gibco, USA) supplemented with 10% foetal bovine serum (FBS) without mycoplasma.

The O-GlcNAcase inhibitors thiamet-G (TMG) and PUGNAc were used at final concentrations of 10 μmol/L and 100 μmol/L for 12 h, respectively.

Overexpression and knockdown lentivirus were purchased from Genechem Company. For lentivirus infection, the HitransG (Genechem, China) was used according to the manufacturer's instructions. The cells were cultured in OPTI-MEM (Gibco, USA) and transduced with lentivirus for 24 h. Then stable cell lines were cultured in DMEM containing 2 μg/ml puromycin (Merck Millipore, Germany) for 1 week.

For transient transfection, attractene reagent (QIAGEN, Germany) was used according to the manufacturer's instructions. The SIX1 and OGT siRNAs were purchased from Genechem China, and the sequences were as follows: SIX1 siRNA1: 5'CCAACAAGCAGAACCAACUTT3', SIX1 siRNA2: 5'GUCAGCAACUGGUUUAAGATT3', OGT siRNA1: 5'TGAGCAGTATTCCGAGAAA 3', OGT siRNA2: 5'CAATCATTTCATTGATCTT3'.

### Western blotting

Total protein was extracted using RIPA buffer supplemented with protease inhibitor (cat# 539134, Merck Millipore, Germany). Equivalent amounts of protein samples (30 μg) were separated by 10% SDS-PAGE gels and transferred to polyvinylidene fluoride membranes. Then, the primary antibodies were incubated at 4 °C overnight, and the secondary antibodies were incubated for 1 h at room temperature. The primary antibodies used were mouse anti-β-actin (Sigma, USA, cat#A2228), anti-O-GlcNAcylation, anti-CDH1 (Abcam, USA, cat#ab2739, cat#ab217038), rabbit anti-SIX1 (Proteintech, USA, cat#10709-1-AP), anti-GLUT1, anti-HK2, anti-GFAT1, anti-c-myc, anti-cyclin-D1 (Cell Signaling Technology, USA, cat#12939S, cat#2867T, cat#5322S, cat#13987 and cat#2978, respectively). The secondary antibodies used were purchased from Cell Signaling Technology and UltraSignal ECL reagent was purchased from 4A Biotech Co. Ltd. Bands were acquired by BioRad ChemiDoc XRS+ Imaging System and quantified with Image Lab (Bio Rad).

### CCK8 and colony formation assay

For Cell Counting Kit 8(CCK8; Dojindo, Japan) assays, cells were plated into 96-well plates at a concentration of 3000 cells per well. The cells were incubated with CCK8 reagent for 4 h, and cell viability was evaluated by measuring the absorbance at 450 nm in a microplate absorbance reader (Thermo Fisher Scientific, USA) every 24 h for 5 days.

Cells were seeded into 6-well plate (2000 - 3000 per well) for colony formation assay. After 2 weeks, the cells were fixed with absolute ethyl alcohol and dyed with 5% crystal violet. The positive cells turned blue and were counted.

### Subcutaneous xenograft mouse model

The animal experiments were approved by the Animal Ethics Committee of the Fourth Military Medical University (Xi'an, China). BALB/c nu/nu mice were purchased from the Experimental Animal Centre of the Fourth Military Medical University (Xi'an, China). BEL7402 and BEL7404 cells transduced with lentivirus and negative control were injected into the subcutaneous tissue at a dose of 2 × 10^6^/ml (n=5 per group). The tumors were separated and weighed when animals were sacrificed after 4 weeks. The tumor volumes were calculated once a week by the formula: V= (length × width^2^)/2 24.

### Glucose uptake assay

The Glucose Uptake Assay Kit was used to determine glucose uptake according to the manufacturer's protocols (cat# J1341, Promega, USA). Briefly, twenty thousand cells were seeded into 96 well plates. The cells were washed with 100 μL PBS and mixed with 50 μL of the prepared 1 mM 2DG per well. After brief shaking, the cells were incubated for 10 minutes at room temperature. Then, 25 μL of Stop Buffer, 25 μL of Neutralization Buffer, 100 μL of 2DG6P Detection Reagent were successively added, followed by brief shaking to mix. Luminescence was recorded using 0.3 s integration on a luminometer. The results were normalized to each control group to calculate relative changes in glucose uptake.

### Real-time PCR

Total RNA was isolated using the mRNA isolation kit (cat# 74134, Qiagen, Germany) and the final RNA samples were stored at -80°C. 1 μg of total RNA were used for cDNAs synthesis using a cDNA Synthesis Kit (Code No. 6215A, Takara, Japan). The relative amount of mRNA was determined with gene-specific primers. The β-actin was used as internal control for mRNAs assays. All steps were performed according to the manufacturer's protocol. The primer sequences for qPCR were showed as Supplementary Table S1.

### Immunoprecipitation

Cell samples were collected with IP lysis buffer (prod#87787, Thermo Scientific, USA) containing protease inhibitor (cat#539134, Merck Millipore, Germany). Then the samples were ultrasonicated for 3 s three times and incubated with the antibodies against SIX1 (cat#12891, Proteintech, USA), OGT (cat# D1D8Q, Abcam, USA), O-GlcNAcylation (cat#ab2739, RL2, Abcam, USA), for 1 h at 4 °C. Then 200 μL magnetic beads (cat# LSKMAGG02, Merck Millipore, Germany) were added to the samples at 4 °C overnight. The beads were washed with PBS for three times and were boiled for further western blotting.

### Ubiquitination test

An ubiquitination detection Kit (Cat# BK161, Cytoskeleton, USA) was used according to the assay protocol. Cells were cultured in 150 cm^2^ plates and lysed after treatment with proteasome inhibitor (30 nmol/L, cat# 539134, MedChemexpress, USA) and deubiquitination inhibitor (1:100, Part# NEM09BB, N-ethylmaleimide & TPEN, Cytoskeleton, USA). Then the supplied filter plunger was used to completely compress the filter and collect the lysate flow through. Aliquots 20 μL of ubiquitination affinity bead suspension were added to each sample to detect the ubiquitination in the samples. In addition, a small amount of lysate (20 μL) was saved to run as a western input lysate control. The beads were washed with wash buffer for three times and eluted with elution buffer to obtain the samples. The enriched protein population was then analyzed by standard western blot procedures.

### Plasmid construction and site directed mutagenesis

Wild type SIX1 plasmid was purchased from Genechem Company using the GV141 vector. Site-directed mutants (Thr276, Ser225 and Thr252 mutated to alanine) were performed by PCR using mutagenic oligonucleotides and mutagenized plasmids were checked by sequencing. The vector, primers and sequences information are shown in Supplementary Table S2.

### Statistical analysis

All *in vitro* experiments were performed three times. Data were presented as the mean ± S.E.M. and were compared between two groups by Student's unpaired t-test. The comparisons between the control group and several experimental groups were performed by one-way ANOVA. The overall survival (OS) of patients and comparisons were plotted using the Kaplan-Meier method. *p* < 0.05 was considered statistically significant.

## Results

### SIX1 is up-regulated and predicts poor survival of HCC

**Figure 1:**
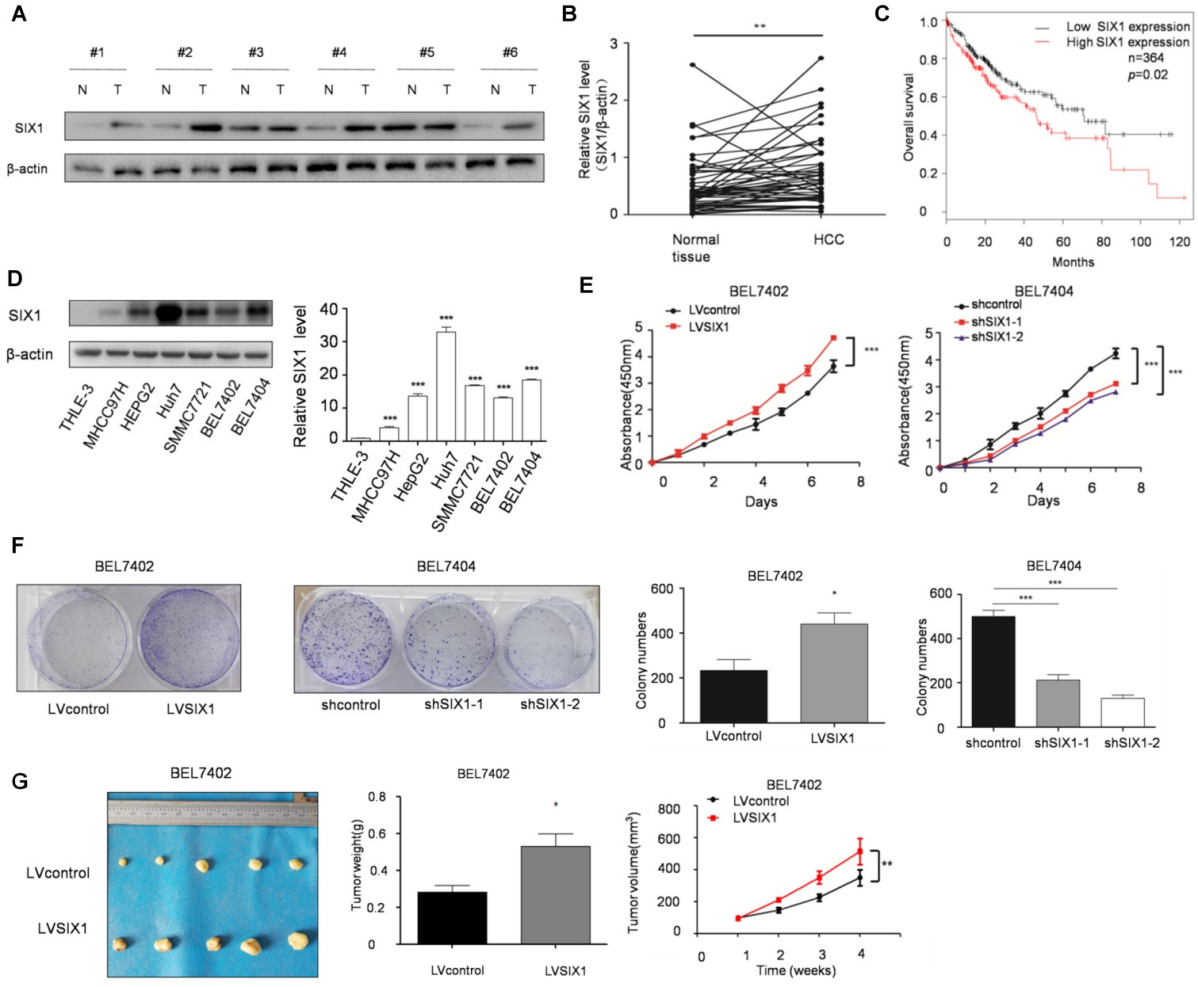
** The expression and function of SIX1 in hepatocellular cancer. A** The level of SIX1 was analyzed in paired HCC specimens and their peritumoral tissues by western blot. **B** Quantitative analysis of SIX1 expression in paired HCC tumor tissues and normal tissues. **C** Overall survival (OS) curve of HCC patients in correlation with the expression of SIX1 using Kaplan-Meier Plotter database (n=364).** D** Western blot and quantitative analysis of SIX1 expression using a panel of HCC cell lines and the immortal liver cell line THLE3.** E and F** Analysis of cell growth using CCK8 (E) and colony formation (F) assays upon manipulation of SIX1. BEL7402 and BEL7404 cells were transduced with a SIX1 overexpression or knockdown lentivirus, respectively. **G** Tumors weights of nude mice were measured after four weeks at the experimental endpoint. **H** Plot of tumor volumes in nude mice measured every week (The data from B, F (left panel), G were analyzed by Student's t-test, data from D and F (right panel) were analyzed by one-way ANOVA and data from E and H were analyzed by two-way ANOVA. *p < 0.05, **p < 0.01, ***p < 0.001).

The level of SIX1 in HCC tissues was examined by Western blot. The results showed that SIX1 expression was higher in HCC tissue than in normal tissues (76%, 38 of 50, based on a 1.2 fold change) (Figure 1A and B). In addition, we observed a significantly worse outcomes in patients with high expression of SIX1, as the median survival was 46.2 months in the high expression group and 71 months in the low expression group (*p* < 0.05, Figure 1C). Next, we analyzed SIX1 expression levels using a panel of HCC cell lines and the immortal liver cell line THLE3. The results with cell lines also confirmed that the protein level of SIX1 was increased in HCC cells compared with the normal control (Figure 1D). After that, CCK8 and colony formation assay were performed to determine cell growth and survival ability, which could reflect proliferation capacity in stable SIX1 manipulation cell line. The efficiency of SIX1 manipulation in BEL7402 and BEL7404 cells was confirmed by western blot (Supplementary Figure S1A). The proliferating effect of SIX1 on proliferation was confirmed by the increased growth of cells and colony formation efficiency in cells with higher SIX1 levels compared to cells with lower SIX1 levels (Figure 1E and F). Moreover, an *in vivo* test also confirmed that overall tumor weight and volume were also increased following SIX1 upregulation (Figure 1G and H, Supplementary Figure S1B and S1C). Collectively, these results suggested that increased SIX1 level promoted the proliferation of HCC.

### SIX1 activates the HBP pathway and increases O-GlcNAcylation levels in HCC cells

**Figure 2:**
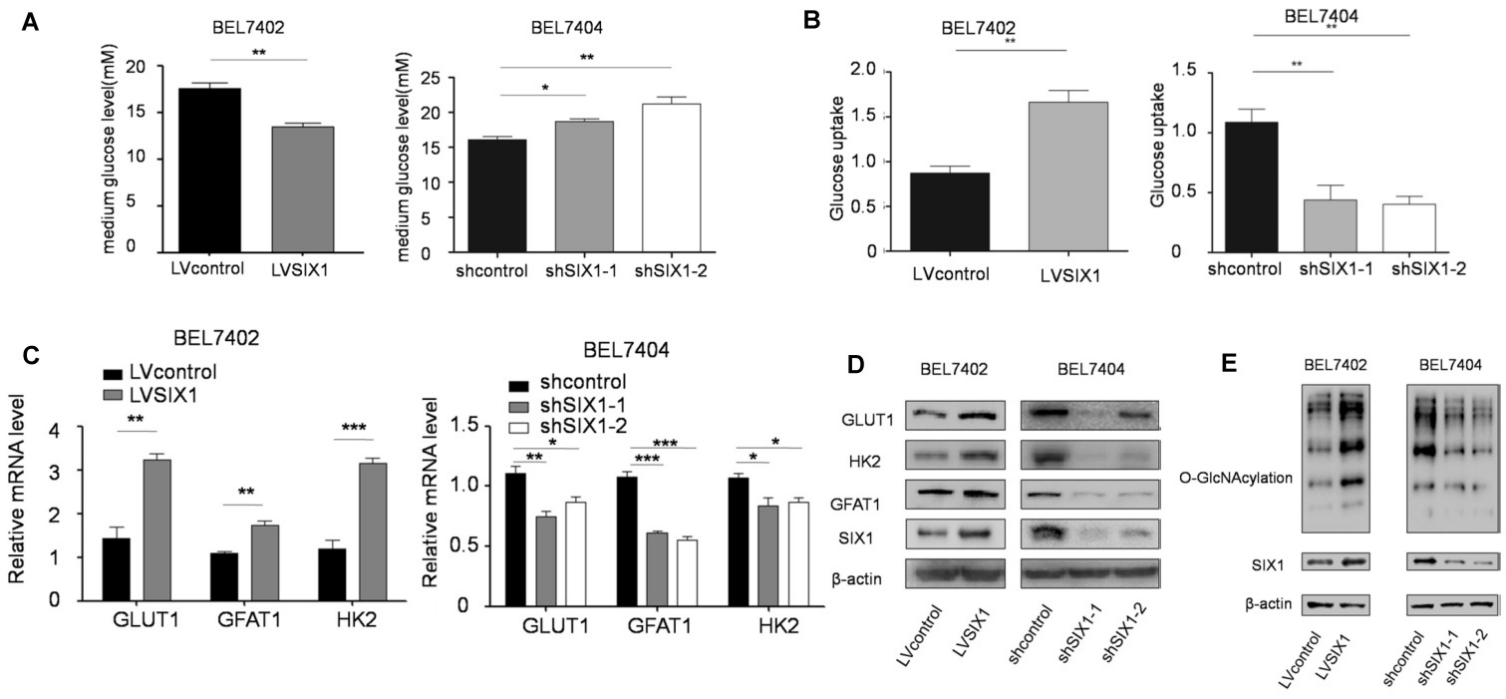
** SIX1 activates the HBP pathway and increases O-GlcNAcylation in HCC.** BEL7402 and BEL7404 cells were transduced with SIX1 overexpression or knockdown lentivirus, respectively. **A** The glucose levels were measured in the cell culture media of cells with SIX1 overexpression or knockdown **B** Glucose uptake levels were determined in the cell line with different SIX1 expressions. **C** The mRNA levels of GLUT1, HK2 and GFAT1 were analyzed with qPCR in cells with SIX1 overexpression or knockdown. **D** GLUT1, HK2 and GFAT1 were analyzed by western blotting in cells with SIX1 overexpression or knockdown. **E** O-GlcNAcylation was analyzed by western blot in HCC cells with SIX1 knockdown or overexpression. (The data were analyzed by Student's t-test (BEL7402) and one-way ANOVA (BEL7404).*p < 0.05, **p < 0.01, ***p < 0.001).

The HBP is shunted out of glycolysis and controls a subsequent important posttranslational modification, O-GlcNAcylation 25. There is mounting evidence that HBP and OGT underscore cancer cell proliferation 26 and that O-GlcNAcylation fluctuates through cell cycle 27. We have demonstrated the role of SIX1 in HCC proliferation, and there is the solid evidence that SIX1 can promote the Warburg effect and increase the expression of several glycolytic genes. We next tested whether there is a connection between SIX1, the Warburg effect and global O-GlcNAcylation in HCC cells. We first tested glucose levels in the media following culture of control vs SIX1 KD cell lines and found that the glucose level in the medium was increased after SIX1 silencing (Figure 2A). Li et al. demonstrated that GLUT1 is a target of SIX1, which implies that this phenomenon could contribute to altered glucose uptake 14. Glucose uptake assays conducted on SIX1 overexpressing vs SIX1 knockdown cells confirmed that SIX1 could promote the glucose uptake in HCC cells, suggesting that SIX1 could also play a role in the metabolism of HCC cells (Figure 2B). To explore the impact of SIX1 on the HBP pathway in HCC, the enzymes involved in the HBP pathway were examined (Supplementary Figure S2A). We found the elevated expression of 3 glycolysis-related genes (GLUT1, GFAT1, and HK2) in SIX1-overexpressing cells using real time PCR (Figure 2C). The protein levels of GLUT1, GFAT1 and HK2 showed a similar trend as the mRNA results (Figure 2D). In addition, we investigated the O-GlcNAcylation levels in HCC cells with different levels of SIX1 expression. The results showed that there was an increased O-GlcNAcylation level in SIX1-overexpressing cell compared to control cells (Figure 2E), indicating that SIX1 promotes the O-GlcNAcylation levels in HCC. Conversely, SIX1 silencing reduced O-GlcNAcylation levels in HCC. To investigate whether increased O-GlcNAcylation is due to the activation of the whole pathway or the OGT or OGA enzyme alone, we next tested the OGA and OGT protein levels, and Glutamine: fructose-6-phosphate amidotransferase (GFAT) activity, the enzyme that catalyzes the first and rate-limiting step in the hexosamine biosynthesis pathway, in the established cell line. The results showed that GFAT activity fluctuated (Supplementary Figure S2B), while OGT and OGA protein levels remained unchanged (Supplementary Figure S2C). These results demonstrate that OGT and OGA protein levels were not significantly influenced by SIX1, which indicates that the activation of the HBP pathway could be the main reason for the increased O-GlcNAcylation level in HCC.

### Upregulation of O-GlcNAcylation promotes the progression of HCC

**Figure 3:**
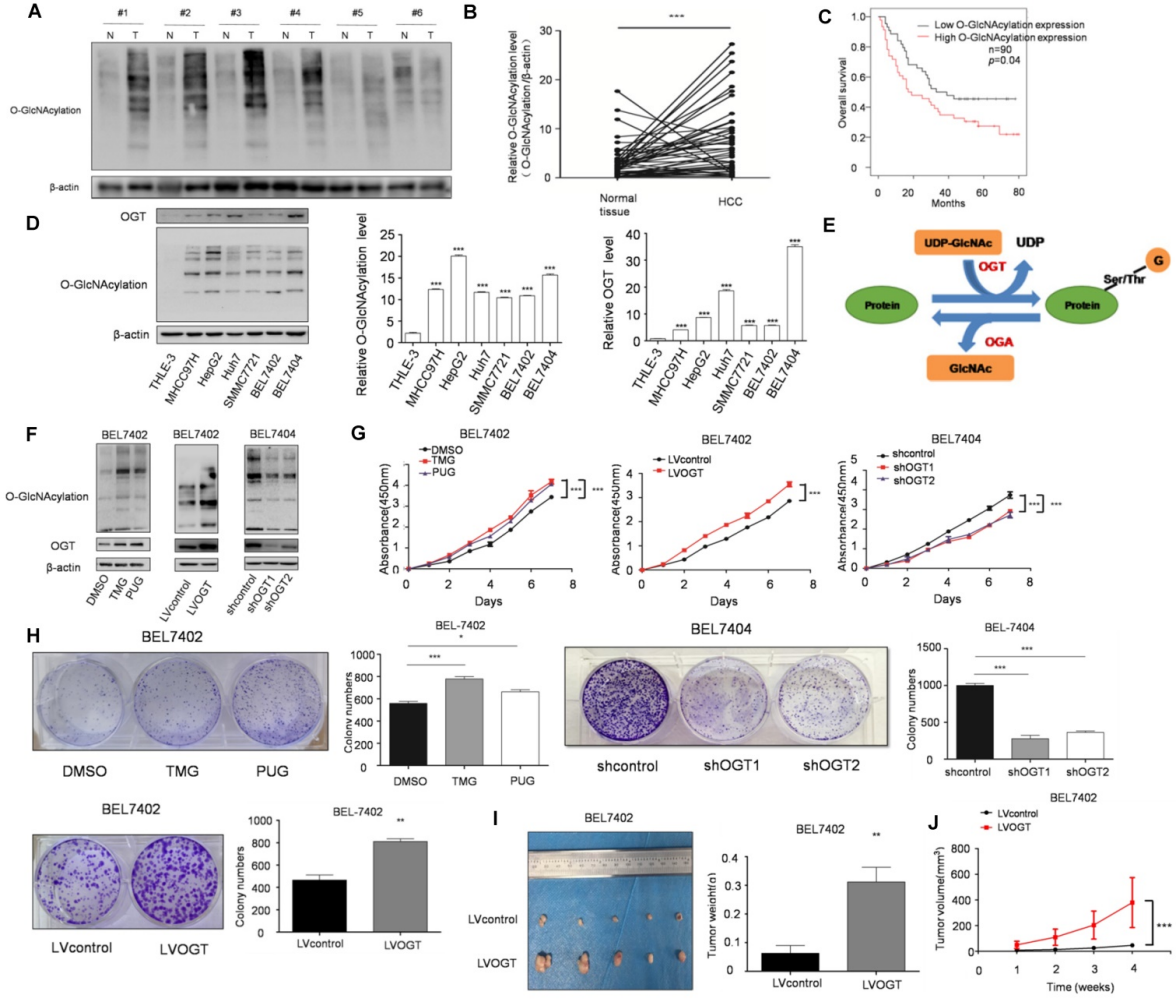
** SIX1 promotes HCC proliferation by regulating O-GlcNAcylation. A** The level of total O‐GlcNAcylation was analyzed in paired HCC specimens and their peritumoral tissues by western blot. **B** Quantitative analysis of O‐GlcNAcylation expression in paired HCC tumor tissues and normal tissues. **C** Overall survival (OS) curve of HCC patients in correlation with expression of O-GlcNAcylation was analyzed using the IHC scores of a tissue microarray analysis (n=90). **D** Western blot and quantitative analysis of O-GlcNAcylation expression using a panel of HCC cell lines and the immortal liver cell line THLE3. **E** Schematic diagram of O-GlcNAcylation. **F** O-GlcNAcylation expression of BEL7402 or BEL7404 cells upon modification of O-GlcNAcylation using OGA inhibitors (TMG and PUGNAc) or OGT lentivirus was measured. **G and H** Analysis of cell growth using CCK-8 (G) and colony formation (H) assay upon modification of O-GlcNAcylation using OGA inhibitors (TMG and PUGNAc) or OGT lentivirus. **I** Tumors weights of nude mice were measured after four weeks at the experimental endpoint. **J** Plot of tumor volumes in nude mice measured every week. (The data from B, H (down panel) and I were analyzed by Student's t-test, data from H (up panel) and D were analyzed by one-way ANOVA and data from G and J were analyzed by two-way ANOVA.*p < 0.05, **p < 0.01, ***p < 0.001).

O-GlcNAcylation, as a link between metabolism and posttranslational modification, has been revealed to play an important role in various cancers. Moreover, we found that SIX1 could increase the O-GlcNAcylation level in HCC cells. To determine whether SIX1 promotes the development of HCC by upregulating O-GlcNAcylation, we further explored the role of O-GlcNAcylation in HCC. Our results showed that the level of O-GlcNAcylation was significantly increased in hepatocellular cancer tissues compared with normal tissues (Figure 3A and B). Moreover, patients with high O-GlcNAcylation expression in the tumors had a worse prognosis than those with low expression (*p* < 0.05, Figure 3C). The results in cell lines also confirmed that O-GlcNAcylation expression levels were significantly higher in HCC cell lines than in the immortalized liver cell line (Figure 3D). Furthermore, we manipulated O-GlcNAcylation levels through its enzymes OGT and OGA (Figure 3E). As shown in Figure 3F, OGA inhibitors (TMG and PUGNAc) and OGT lentivirus showed a significant capacity to manipulate O-GlcNAcylation. By using CCK8 and colony formation assays, we found that elevated levels of O-GlcNAcylation promoted HCC cell growth (Figure. 3G and H). In addition, mice in the hyper O-GlcNAcylation group displayed larger tumors than their counterparts (Figure 3I and J). In summary, these data suggest that high O-GlcNAcylation expression is critical for HCC cell proliferation.

### SIX1 is directly modified by O-GlcNAcylation in HCC

**Figure 4:**
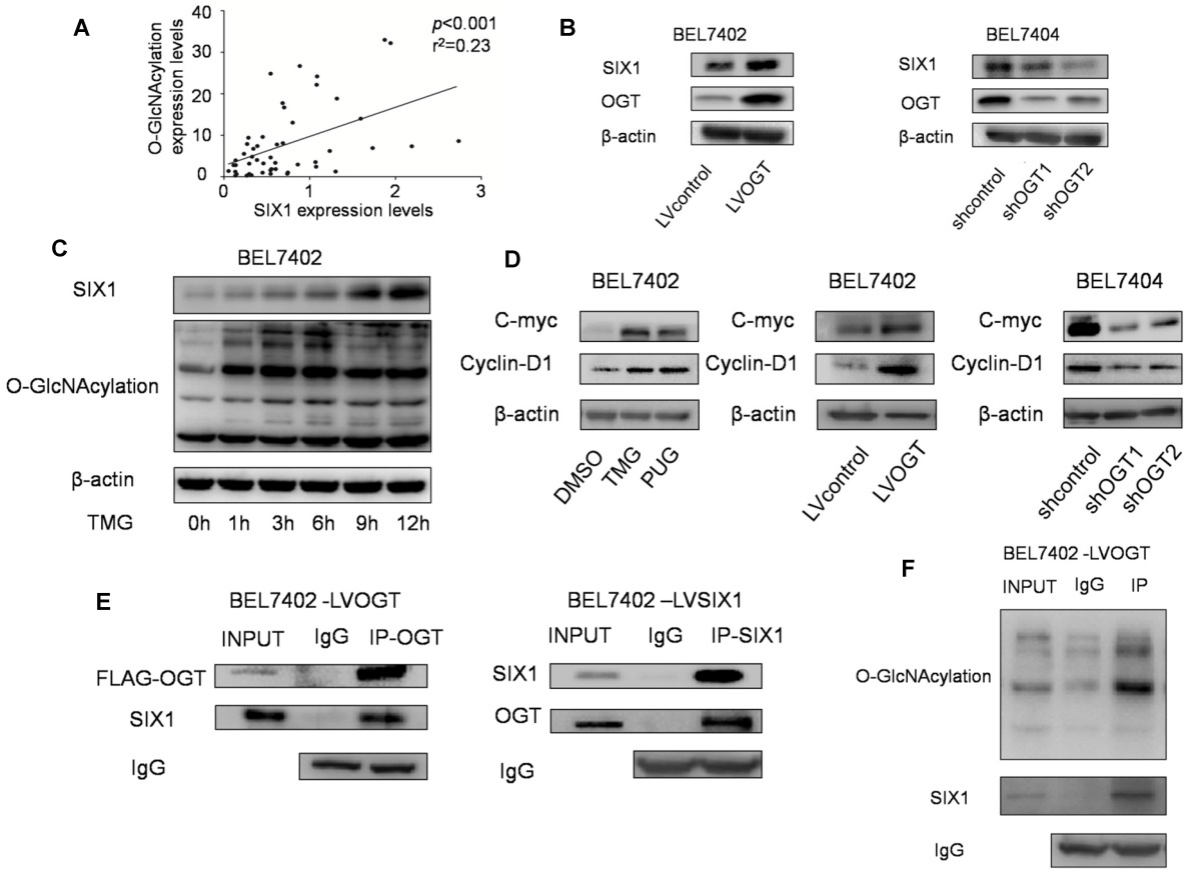
** O-GlcNAcylation has reverse effect on SIX1 expression in HCC. A** Relationship between O-GlcNAcylation and SIX1 expression levels in fifty HCC samples. BEL7402 and BEL7404 were treated with OGA inhibitors or transduced with OGT lentivirus (overexpression or knockdown) as indicated. **B** SIX1 expression levels were analyzed by western blotting in established HCC cell lines with different manipulation of O-GlcNAcylation.** C** SIX1 expression in BEL7402 cells treated with an OGA inhibitor (TMG) at the indicated times. **D** The direct target genes of SIX1 were analyzed in established HCC cell lines. **E** Immunoprecipitation with anti-OGT antibody followed by western blotting with anti-SIX1 antibody. Same experiment was performed when SIX1 was immunoprecipitated and immunoblotted with anti-OGT antibody. **F** O-GlcNAcylation modified protein immunoprecipitated from cell extracts was analyzed by immunoblotting for anti-SIX1.

A previous study showed that SIX1 can be regulated by posttranslational modifications and we subsequently investigated whether O-GlcNAcylation could also modulate SIX1. In our tests, we observed a statistically significant positive correlation between SIX1 expression and O-GlcNAcylation levels in primary HCC samples (Figure 4A). Strikingly, we also found that SIX1 was increased following with the overexpression of OGT, and reduced in response to OGT knockdown (Figure 4B). In addition, we investigated the SIX1 levels after stimulation of O-GlcNAcylation by using the OGA inhibitor TMG at a series of time points. Over time, the levels of SIX1 were increased following the upregulation of O-GlcNAcylation (Figure 4C). Furthermore, we examined some classical oncogenic factors regulated by SIX1. We found that these factors were upregulated along with hyper O-GlcNAcylation levels (Figure 4D). To determine whether SIX1 could indeed be modified by O-GlcNAcylation, an immunoprecipitation experiment was performed. The results showed that SIX1 could interact with OGT in HCC cells (Figure 4E). In addition, total O-GlcNAcylation modified proteins were purified from cells and examined by immunoblotting using an anti-SIX1 antibody. The results confirmed that SIX1 could be O-GlcNAcylated (Figure 4F). The mass spectrum analysis of O-GlcNAcylation protein also showed that SIX1 was among the 207 total potential proteins (Supplementary Table S3). Based on these results, we concluded that SIX1 can directly bind to OGT and can be modified by O-GlcNAcylation.

### O-GlcNAcylation enhances SIX1 levels by inhibiting ubiquitination

**Figure 5:**
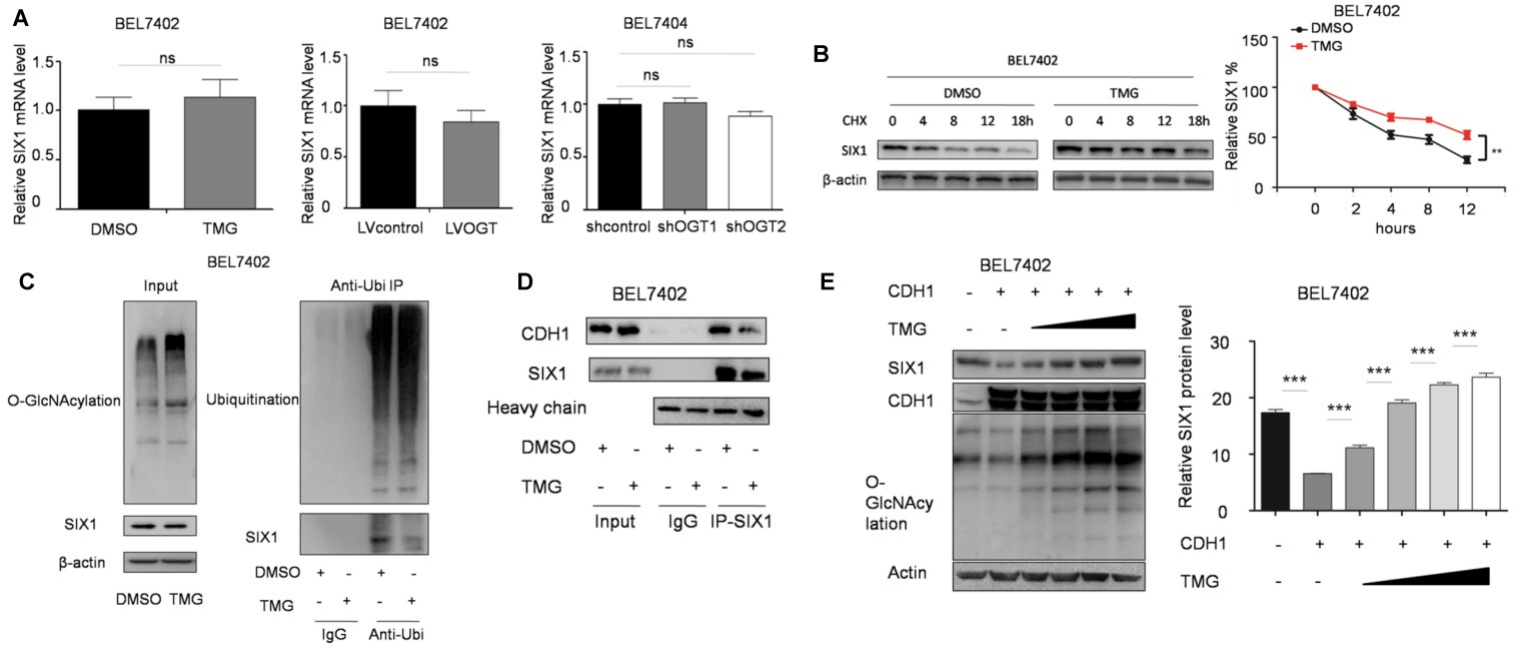
** O-GlcNAcylation stabilizes SIX1 via CDH1. A** The mRNA levels of SIX1 were analyzed in HCC cell lines as indicated. **B** Levels of SIX1 were determined by western blotting in BEL7402 cells treated with CHX (10 μg/ml) for the indicated times. **C** Ubiquitination of SIX1 was examined in BEL7402 cells after OGA inhibitor treatment. MG132 and NEM were used to inhibit proteasome and deubiquitination, respectively. **D** CDH1 was detected in purified SIX1 immunoprecipitation samples from BEL7402 treated with DMSO or TMG. E SIX1 expression was examined in BEL7402 CDH1-overexpressing cells with an increasing amount of TMG. (The data from right panel of A and **E** were analyzed by one-way ANOVA, data from B were analyzed by two-way ANOVA, and the rest of the statistics were analyzed by Student's t-test. ns: no significance, *p < 0.05, **p < 0.01, ***p < 0.001).

We subsequently investigated how O-GlcNAcylation regulates the SIX1 expression level. We found that that O-GlcNAcylation had no influence on the mRNA levels of SIX1 (Figure 5A). SIX1 has been reported to be degraded through the ubiquitin-proteasome pathway 23, and we hypothesized that O-GlcNAcylation could inhibit the ubiquitination of SIX1. To test this hypothesis, we analyzed the degradation of SIX1 expression by using cycloheximide. The results showed that the SIX1 degradation was slowed by the hyper O-GlcNAcylation, which suggested that O-GlcNAcylation may regulate the expression of SIX1 by affecting its ubiquitination (Figure 5B). We further analyzed the ubiquitination levels of SIX1 after the O-GlcNAcylation levels were altered. In the ubiquitination test, we used proteasome and deubiquitination inhibitors to accumulate and increase the chances of detecting rapid and transient modifications. We found that when the deubiquitin and proteasome inhibitors were used to block the ubiquitination degradation process, the previously observed increase of SIX1 along with the increase in O-GlcNAcylation was blocked (Figure 5C left panel). Then we purified the total ubiquitinated proteins and found that SIX1 ubiquitination levels were lower in the TMG treated group than in the control group under similar overall ubiquitination conditions (Figure 5C right panel). CDC20 homologue 1 (CDH1) has been reported to be an ubiquitin E3 ligase that mediates SIX1 degradation 23. To test whether that hyper O-GlcNAcylation could affect SIX1 degradation through CDH1, we purified the SIX1 protein in cells with different O-GlcNAcylation levels and immunobloted with anti-CDH1 antibody. The results showed that the co-immunoprecipitation of CDH1 with SIX1 was decreased in the hyper O-GlcNAcylation group compared with the control group despite the similar levels in the input samples (Figure 5D). Furthermore, SIX1 expression was examined in BEL7402 cells overexpressing CDH1 with an increasing amount of TMG treatment. The results showed that hyper O-GlcNAcylation reversed CDH1-induced SIX1 degradation (Figure 5E). Collectively, our results suggested that O-GlcNAcylation of SIX1 could inhibit the ubiquitination-dependent degradation of SIX1 through CDH1.

### The O-GlcNAcylation site mutant of SIX1 Thr276 decreased its stability

**Figure 6:**
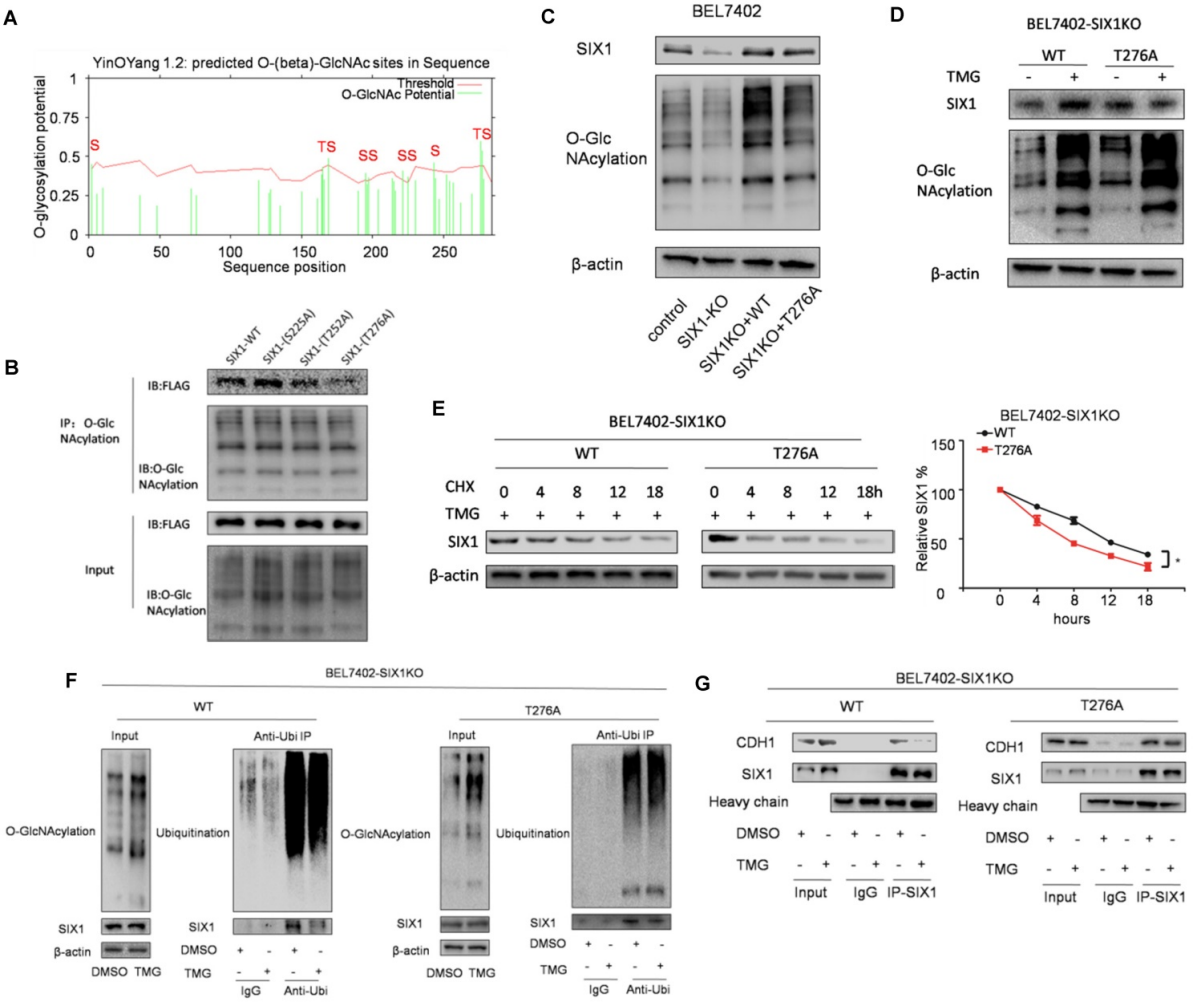
** Mutation in Thr276 decreases the O-GlcNAcylation of SIX1. A** The O-GlcNAcylation sites of SIX1 predicted by the YinOYang 1.2 server (www.cbs.dtu.dk/services/YinOYang) are shown with a black arrowhead at the top. The green vertical lines show the potential O-GlcNAc-modified Ser/Thr residues, and the red horizontal wavy line indicates the threshold for modification potential. **B** The level of SIX1 protein in IP-O-GlcNAcylation samples from BEL7402 cells transfected with plasmids expressing wild-type or O-GlcNAcylation site mutant was analyzed by western blot. **C** The establishment of SIX1 WT and T276A cells using the CRISPR-Cas9 system and subsequent ectopic expression. **D** The wild-type and T276A SIX1 expression was detected in BEL7402-SIX1KO cells transfected with WT or T276A SIX1-expressing plasmids under the treatment of DMSO or TMG. **E** Levels of wild-type and T276A SIX1 were determined by western blot in BEL7402-SIX1KO cells transfected with WT or T276A SIX1-expressing plasmids after TMG and CHX treatment for the indicated times. **F** Ubiquitination of wild-type and T276A SIX1 were examined in BEL7402-SIX1KO cells transfected with WT or T276A SIX1-expressing plasmids. MG132 and NEM were used to inhibit proteasome and deubiquitination, respectively. **G** CDH1 was detected in purified wild-type and T276A SIX1 IP samples in BEL7402-SIX1KO cells transfected with WT or T276A SIX1-expressing plasmids. (The data from E were analyzed by two-way ANOVA, *p < 0.05, **p < 0.01, ***p < 0.001).

To determine the location of the O-GlcNAcylation site(s) on SIX1, we used two different online databases (YinOYang 1.2 Server and OGTSite) to predict potential O-GlcNAcylation sites in SIX1. The results showed that SIX1 was predicted to have multiple potential O-GlcNAcylation sites (Figure 6A). The results showed that Thr276, Ser225 and Thr252 had the highest probability of being modified by O-GlcNAcylation. To further investigate the potential sites of SIX1 O-GlcNAcylation, individual site mutants of SIX1 (T225A, T252A, T276A) were expressed in BEL7402 cells. We observed that the T276A mutation had most dramatic reduction of O-GlcNAcylation via immunoprecipitation with the O-GlcNAcylation antibody (Figure 6B), confirming that Thr276 in SIX1 is a potential O-GlcNAcylation site. To investigate the potential importance of T276 O-GlcNAcylation, we established BEL7402-SIX1KO cell lines by depleting endogenous SIX1 using the CRISPR-Cas9 system. Then we rescued BEL7402-SIX1KO with ectopic expression of SIX1 WT or SIX1 T276A and used these cells in following experiments. We found that mutant SIX1 could also reverse the level of O-GlcNAcylation similar to wild -type SIX1 in HCC cells (Figure 6C). After TMG treatment, the wild-type SIX1 level was significantly higher than the mutant level (Figure 6D). In addition, wild-type SIX1 but not the mutant, was found to display a longer half-life under hyper O-GlcNAcylation (Figure 6E). Furthermore, we found that the ubiquitination of T276A SIX1 could not be inhibited by hyper O-GlcNAcylation (Figure 6F, Supplementary Figure S3), suggesting that Thr276 is important for the ubiquitination of SIX1. To test whether O-GlcNAcylation at Thr276 affects CDH1 binding to SIX1, we performed co-IP experiments in the WT and mutant groups. We found that CDH1 was dissociated from complexes pulled down by the anti-SIX1 antibody when the WT SIX1 group was stimulated by TMG. However, the inhibitory effect of hyper O-GlcNAcylation was not significant when Thr276 was mutated (Figure 6G). In addition, in order to exclude the cell specificity, we used another cell line HepG2 to confirm these conclusions (Supplementary Figure S4). Together, these data strongly indicated that Thr276 was the potential O-GlcNAcylation sites of SIX1 and that O-GlcNAcylation at Thr276 protects SIX1 from degradation.

### O-GlcNAcylation is necessary for the tumor-promoting effect of SIX1

**Figure 7:**
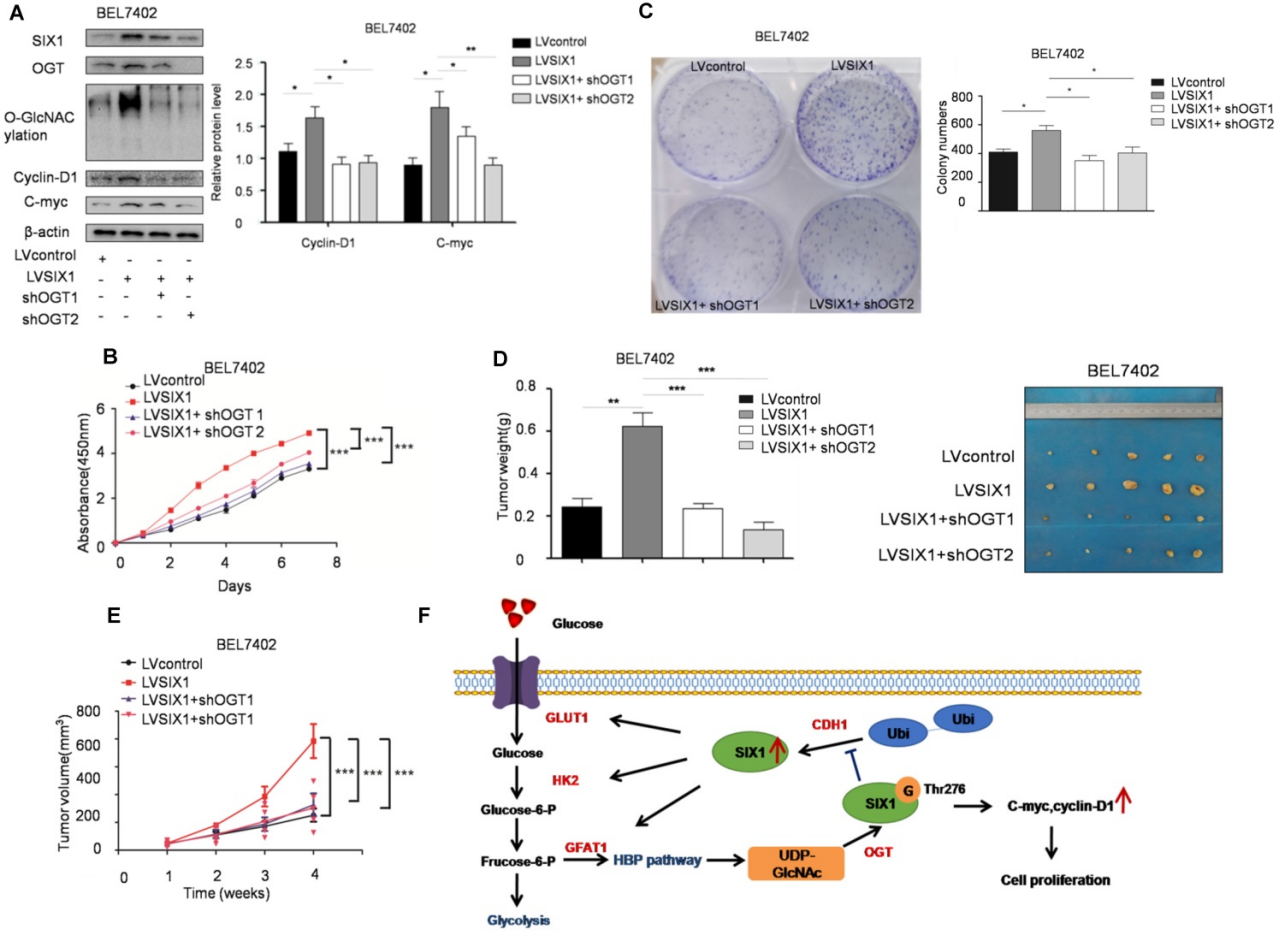
** Depletion of OGT reverses the tumor-promoting effect of SIX1 overexpression. A** SIX1 downstream factor protein levels and quantitative analysis data in BEL7402 treated as indicated were examined by western blot**. B and C** CCK8 (B) and Colony formation (C) assays were performed in BEL7402 cells infected with the indicated lentivirus. **D** Tumors weights of nude mice were measured after four weeks at the experimental endpoint. **E** Plot of tumor volumes in nude mice was measured every week. **F** Schematic model of O-GlcNAcylation and SIX1 in HCC progression. The sign “G” indicates the O-GlcNAc and “Ubi” indicates the ubiquitin. (The data from A, C and D were analyzed by one-way ANOVA, and data from B, E were analyzed by two-way ANOVA. *p < 0.05, **p < 0.01, ***p < 0.001).

To test the role of SIX1 O-GlcNAcylation in HCC, a loss of function experiment was performed. We used a lentivirus shRNA system to evaluate the role of O-GlcNAcylation in HCC development. The results showed that the expression levels of SIX1 target proteins were also reduced in cells with OGT knockdown (Figure 7A). CCK8 and colony formation assays showed that the knockdown of OGT expression could reverse the effect of SIX1 overexpression in cell growth (Figure 7B and C). Importantly, *in vivo* xenograft experiments also demonstrated that the OGT knockdown group has less tumor volume than the control group (Figure 7D and E). In addition, we tested the expression of SIX1, OGT, cyclin-D1, c-myc and the O-GlcNAcylation level in subcutaneous tumors and observed the similar results to the* in vitro* study (Supplementary Figure S5). The results of loss of function assays showed that the tumor-promoting effect of SIX1 on HCC cell progression is dependent on O-GlcNAcylation.

## Discussion

The Warburg effect is the hallmark of cancer cells characterized by a high rate of glycolysis even with a sufficient oxygen supply. SIX1 is reported to be a key transcription factor in the regulation of the Warburg effect 14 and promotes the progression of various cancers. Our study demonstrates the role of SIX1 and further confirms a SIX1/ O-GlcNAcylation feedback loop in HCC (Figure 7F). We show that SIX1 can facilitate tumor growth and further increase HBP gene expression, glucose uptake, and the level of O-GlcNAcylation. Moreover, O-GlcNAcylation of SIX1 at Thr276 protects it against proteasomal degradation and increases protein stability. Our data identified a previously unknown mechanism for the coordination of glucose metabolism and posttranslational modification in HCC.

SIX1 has been implicated in various cancers including HCC 28, 29. It has been reported that SIX1 is overexpressed in HCC tissues and positively associated with venous infiltration, TNM stage and poor overall survival rate 17. Another study further demonstrated that suppression of SIX1 decreases the proliferation and metastatic ability of HCC cells 30. In a recent study, Chen et al. demonstrated that SIX1 could reduce 5-fluorouracil sensitivity in HCC cells 31, suggesting its important role in combined therapy and drug development. Our study also revealed that SIX1 is upregulated and promotes the proliferation in HCC both *in vitro and in vivo*. We found that nearly 76% (38 out of 50) of HCC samples expressed higher SIX1 protein than the paired normal tissues, which was consistent with a previous report 17.

Several previous studies have reported that the molecular mechanisms by which SIX1 exerts its abilities in HCC are mediated by attenuating the stemness of HCC cells, and regulating p53, MMP9 32, 33. However, as a glycolysis regulator, whether metabolism is a mediator of SIX1 in HCC progression has not yet been explored. Because of the close relationship between SIX1 and glycolysis, we hypothesized that SIX1 may also be correlated with glycolysis in HCC. In our study, we confirmed the role of SIX1 in glucose metabolism and further found its positive relationship with the HBP. We demonstrate that SIX1 could enhance the expression level of HBP genes and subsequent O-GlcNAcylation, which dynamically links the cell metabolism with posttranslational modification. However, the exact regulatory mechanism of SIX1 and O-GlcNAcylation still need further investigation.

As a linkage between metabolism and cellular processes, O-GlcNAcylation can modify a large number of proteins and is believed to play a significant role in cancer development through posttranslational modifications 34-36. A previous study by our research group showed that O-GlcNAcylation was higher in gastric cancer tissues than in adjacent normal tissues 37. In hepatocellular cancer, Xu and Zhang et al. found that OGT and O-GlcNAcylation were higher in HCC cell lines and liver cancer tissues than in their respective normal counterparts, respectively 38, 39. Zhu et al. demonstrated that O-GlcNAcylation plays a role in tumor recurrence of hepatocellular carcinoma following liver transplantation 40. In our study, the results also show that O-GlcNAcylation is significantly increased in the HCC samples. Through further *in vitro* and *in vivo* analysis, we found that the O-GlcNAcylation significantly promoted the proliferation of HCC cells.

O-GlcNAcylation has been demonstrated to be an important mechanism in the response to aberrant metabolic signals and promotes cancer progression. It has been proven that phosphofrutokinase 1, pyruvate kinase and phosphoglycerate kinase 1 are regulated by O-GlcNAcylation 41-43. Our previous study showed that O-GlcNAcylation of CD36, a regulator of fatty acid transport, could promote gastric cancer metastasis 44. These reports suggest that O-GlcNAcylation could participate in regulating many biological factors. However, whether SIX1, a major regulator of glycolysis in cancer, can be regulated by O-GlcNAcylation was not clear. In the present study, we found that SIX1 was positively correlated with O-GlcNAcylation in HCC tissues. Further immunoprecipitation and mass spectrometry studies demonstrated that SIX1 could directly modified by O-GlcNAcylation. We also find that Thr276 is the potential O-GlcNAcylation site of SIX1. By O-GlcNAcylation, the ubiquitination of SIX1 can be prevented, therefore promoting the proliferation of HCC cells. Additionally, this phenotype was rescued when OGT was downregulated in these cells, suggesting that it was the modification that was responsible for the malignant progression. Although the evidence indicates that Thr276 is the potential O-GlcNAcylation site and O-GlcNAcylation of the mutant T276A-SIX1 is significantly decreased, SIX1 O-GlcNAcylation was not completely abolished, and we still need to map the site of O-GlcNAcylation on SIX1 using mass spectrometry for the direct proof or the identification of other O-GlcNAcylation sites. It is expected that further studies will reveal a broader role of O-GlcNAcylation in regulating cell metabolism.

In addition, there is ample evidence for a role of SIX1 in cancer metastasis and EMT. Zhu et al. demonstrated that SIX1 enhanced Vimentin expression at the transcriptional level by directly binding to the promoter region of Vimentin, thereby promoting gastric cancer cell migration and invasion 45. Therefore, in the future, elucidating the function of O-GlcNAcylation in SIX1's role in metastasis will continue to expand the network of SIX1 and O-GlcNAcylation.

Taken together, we present a new regulatory pathway of the SIX1/ O-GlcNAcylation feedback loop in HCC. Upregulated SIX1 increases the glucose uptake and further increases the level of O-GlcNAcylation in HCC cells. In addition, hyper O-GlcNAcylation inhibits the ubiquitination of SIX1 and sustains SIX1 and its downstream factors in high expressions, which further facilitates the malignant phenotype of HCC. This study indicates that SIX1/O-GlcNAcylation might be a significant pathway for tumor proliferation and a promising therapeutic target for treating HCC.

## Supplementary Material

Supplementary figures and tables.

## Abbreviations

SIX1: sine oculis homeobox homolog 1
HCC: hepatocellular carcinoma
O-GlcNAcylation: o-linked β-N-acetylglucosaminylation
CCK8: Cell Counting Kit 8
OGT: O-GlcNAc transferase
OGA: O-GlcNAcase
HBP: hexosamine biosynthetic pathway
TMG: thiamet-G

## References

[1] Koppenol WH, Bounds PL, Dang CV (2011). Otto Warburg's contributions to current concepts of cancer metabolism. Nat Rev Cancer.

[2] DeBerardinis RJ, Lum JJ, Hatzivassiliou G, Thompson CB (2008). The biology of cancer: metabolic reprogramming fuels cell growth and proliferation. Cell Metab.

[3] Marshall S (2006). Role of insulin, adipocyte hormones, and nutrient-sensing pathways in regulating fuel metabolism and energy homeostasis: a nutritional perspective of diabetes, obesity, and cancer. Sci STKE.

[4] Lin SH, Liu T, Ming X, Tang Z, Fu L, Schmitt-Kopplin P (2016). Regulatory role of hexosamine biosynthetic pathway on hepatic cancer stem cell marker CD133 under low glucose conditions. Sci Rep.

[5] Hart GW, Housley MP, Slawson C (2007). Cycling of O-linked beta-N-acetylglucosamine on nucleocytoplasmic proteins. Nature.

[6] Vosseller K, Wells L, Hart GW (2001). Nucleocytoplasmic O-glycosylation: O-GlcNAc and functional proteomics. Biochimie.

[7] Singh JP, Zhang K, Wu J, Yang X (2015). O-GlcNAc signaling in cancer metabolism and epigenetics. Cancer Lett.

[8] Ferrer CM, Lu TY, Bacigalupa ZA, Katsetos CD, Sinclair DA, Reginato MJ (2017). O-GlcNAcylation regulates breast cancer metastasis via SIRT1 modulation of FOXM1 pathway. Oncogene.

[9] Yehezkel G, Cohen L, Kliger A, Manor E, Khalaila I (2012). O-linked β-N-acetylglucosaminylation (O-GlcNAcylation) in primary and metastatic colorectal cancer clones and effect of N-acetyl-β-D-glucosaminidase silencing on cell phenotype and transcriptome. J Biol Chem.

[10] de Queiroz RM, Madan R, Chien J, Dias WB, Slawson C (2016). Changes in O-Linked N-Acetylglucosamine (O-GlcNAc) Homeostasis Activate the p53 Pathway in Ovarian Cancer Cells. J Biol Chem.

[11] Caldwell SA, Jackson SR, Shahriari KS, Lynch TP, Sethi G, Walker S (2010). Nutrient sensor O-GlcNAc transferase regulates breast cancer tumorigenesis through targeting of the oncogenic transcription factor FoxM1. Oncogene.

[12] Itkonen HM, Minner S, Guldvik IJ, Sandmann MJ, Tsourlakis MC, Berge V (2013). O-GlcNAc transferase integrates metabolic pathways to regulate the stability of c-MYC in human prostate cancer cells. Cancer Res.

[13] Zhou F, Yang X, Zhao H, Liu Y, Feng Y, An R, Lv X, Li J, Chen B (2018). Down-regulation of OGT promotes cisplatin resistance by inducing autophagy in ovarian cancer. Theranostics.

[14] Li L, Liang Y, Kang L, Liu Y, Gao S, Chen S (2018). Transcriptional Regulation of the Warburg Effect in Cancer by SIX1. Cancer Cell.

[15] Ford HL, Kabingu EN, Bump EA, Mutter GL, Pardee AB (1998). Abrogation of the G2 cell cycle checkpoint associated with overexpression of HSIX1: a possible mechanism of breast carcinogenesis. Proc Natl Acad Sci U S A.

[16] Behbakht K, Qamar L, Aldridge CS, Coletta RD, Davidson SA, Thorburn A (2007). Six1 overexpression in ovarian carcinoma causes resistance to TRAIL-mediated apoptosis and is associated with poor survival. Cancer Res.

[17] Ng KT, Man K, Sun CK, Lee TK, Poon RT, Lo CM (2006). Clinicopathological significance of homeoprotein Six1 in hepatocellular carcinoma. Br J Cancer.

[18] Yu Y, Davicioni E, Triche TJ, Merlino G (2006). The homeoprotein six1 transcriptionally activates multiple protumorigenic genes but requires ezrin to promote metastasis. Cancer Res.

[19] Coletta RD, Christensen K, Reichenberger KJ, Lamb J, Micomonaco D, Huang L (2004). The Six1 homeoprotein stimulates tumorigenesis by reactivation of cyclin A1. Proc Natl Acad Sci U S A.

[20] Wang CA, Jedlicka P, Patrick AN, Micalizzi DS, Lemmer KC, Deitsch E (2012). SIX1 induces lymphangiogenesis and metastasis via upregulation of VEGF-C in mouse models of breast cancer. J Clin Invest.

[21] Ono H, Imoto I, Kozaki K, Tsuda H, Matsui T, Kurasawa Y (2012). SIX1 promotes epithelial-mesenchymal transition in colorectal cancer through ZEB1 activation. Oncogene.

[22] Ford HL, Landesman-Bollag E, Dacwag CS, Stukenberg PT, Pardee AB, Seldin DC (2000). Cell cycle-regulated phosphorylation of the human SIX1 homeodomain protein. J Biol Chem.

[23] Christensen KL, Brennan JD, Aldridge CS, Ford HL (2007). Cell cycle regulation of the human Six1 homeoprotein is mediated by APC(Cdh1). Oncogene.

[24] Naito S, von Eschenbach AC, Giavazzi R, Fidler IJ (1986). Growth and metastasis of tumor cells isolated from a human renal cell carcinoma implanted into different organs of nude mice. Cancer Res.

[25] Slawson C, Copeland RJ, Hart GW (2010). O-GlcNAc signaling: a metabolic link between diabetes and cancer?. Trends Biochem Sci.

[26] Tan EP, Duncan FE, Slawson C (2017). The sweet side of the cell cycle. Biochem Soc Trans.

[27] Liu C, Li J (2018). O-GlcNAc: A Sweetheart of the Cell Cycle and DNA Damage Response. Front Endocrinol (Lausanne).

[28] Kingsbury TJ, Kim M, Civin CI (2019). Regulation of cancer stem cell properties by SIX1, a member of the PAX-SIX-EYA-DACH network. Adv Cancer Res.

[29] Zhao Z, Li L, Du P, Ma L, Zhang W, Zheng L, Lan B, Zhang B, Ma F, Xu B, Zhan Q, Song Y (2019). Transcriptional Downregulation of miR-4306 serves as a New Therapeutic Target for Triple Negative Breast Cancer. Theranostics.

[30] Ng KT, Lee TK, Cheng Q, Wo JY, Sun CK, Guo DY, Lim ZX, Lo CM, Poon RT, Fan ST, Man K (2010). Suppression of tumorigenesis and metastasis of hepatocellular carcinoma by shRNA interference targeting on homeoprotein Six1. Int J Cancer.

[31] Chen K, Wei H, Pan J, Chen Z, Pan D, Gao T (2019). Six1 is negatively correlated with poor prognosis and reduces 5-fluorouracil sensitivity via attenuating the stemness of hepatocellular carcinoma cells. Eur J Pharmacol.

[32] Cheng Q, Ning D, Chen J, Li X, Chen XP, Jiang L (2018). SIX1 and DACH1 influence the proliferation and apoptosis of hepatocellular carcinoma through regulating p53. Cancer Biol Ther.

[33] Zhang Y, Wang S, Liu Z, Yang L, Liu J, Xiu M (2019). Increased Six1 expression in macrophages promotes hepatocellular carcinoma growth and invasion by regulating MMP-9. J Cell Mol Med.

[34] Hanover JA, Krause MW, Love DC (2012). Bittersweet memories: linking metabolism to epigenetics through O-GlcNAcylation. Nat Rev Mol Cell Biol.

[35] Itkonen HM, Urbanucci A, Martin SE, Khan A, Mathelier A, Thiede B (2019). High OGT activity is essential for MYC-driven proliferation of prostate cancer cells. Theranostics.

[36] Sharma NS, Gupta VK, Dauer P, Kesh K, Hadad R, Giri B (2019). O-GlcNAc modification of Sox2 regulates self-renewal in pancreatic cancer by promoting its stability. Theranostics.

[37] Jiang M, Qiu Z, Zhang S, Fan X, Cai X, Xu B (2016). Elevated O-GlcNAcylation promotes gastric cancer cells proliferation by modulating cell cycle related proteins and ERK 1/2 signaling. Oncotarget.

[38] Xu W, Zhang X, Wu JL, Fu L, Liu K, Liu D (2017). O-GlcNAc transferase promotes fatty liver-associated liver cancer through inducing palmitic acid and activating endoplasmic reticulum stress. J Hepatol.

[39] Zhang X, Qiao Y, Wu Q, Chen Y, Zou S, Liu X (2017). The essential role of YAP O-GlcNAcylation in high-glucose-stimulated liver tumorigenesis. Nat Commun.

[40] Zhu Q, Zhou L, Yang Z, Lai M, Xie H, Wu L, Xing C, Zhang F, Zheng S (2012). O-GlcNAcylation plays a role in tumor recurrence of hepatocellular carcinoma following liver transplantation. Med Oncol.

[41] Yi W, Clark PM, Mason DE, Keenan MC, Hill C, Goddard WA 3rd (2012). Phosphofructokinase 1 glycosylation regulates cell growth and metabolism. Science.

[42] Wang Y, Liu J, Jin X, Zhang D, Li D, Hao F (2017). O-GlcNAcylation destabilizes the active tetrameric PKM2 to promote the Warburg effect. Proc Natl Acad Sci U S A.

[43] Nie H, Ju H, Fan J, Shi X, Cheng Y, Cang X (2020). O-GlcNAcylation of PGK1 coordinates glycolysis and TCA cycle to promote tumor growth. Nat Commun.

[44] Jiang M, Wu N, Xu B, Chu Y, Li X, Su S (2019). Fatty acid-induced CD36 expression via O-GlcNAcylation drives gastric cancer metastasis. Theranostics.

[45] Zhu Z, Rong Z, Luo Z, Yu Z, Zhang J, Qiu Z (2019). Circular RNA circNHSL1 promotes gastric cancer progression through the miR-1306-3p/SIX1/vimentin axis. Mol Cancer.

