# Supplementary material for: O-GlcNAcylation of SIX1 enhances its stability and promotes Hepatocellular Carcinoma Proliferation
Source: Theranostics. 2020 Aug 2;10(21):9830–42. doi: 10.7150/thno.45161 (PMC7449927; doi:10.7150/thno.45161)
Supplement: Supplementary file 1 — Supplementary figures and tables. [file thnov10p9830s1.pdf]

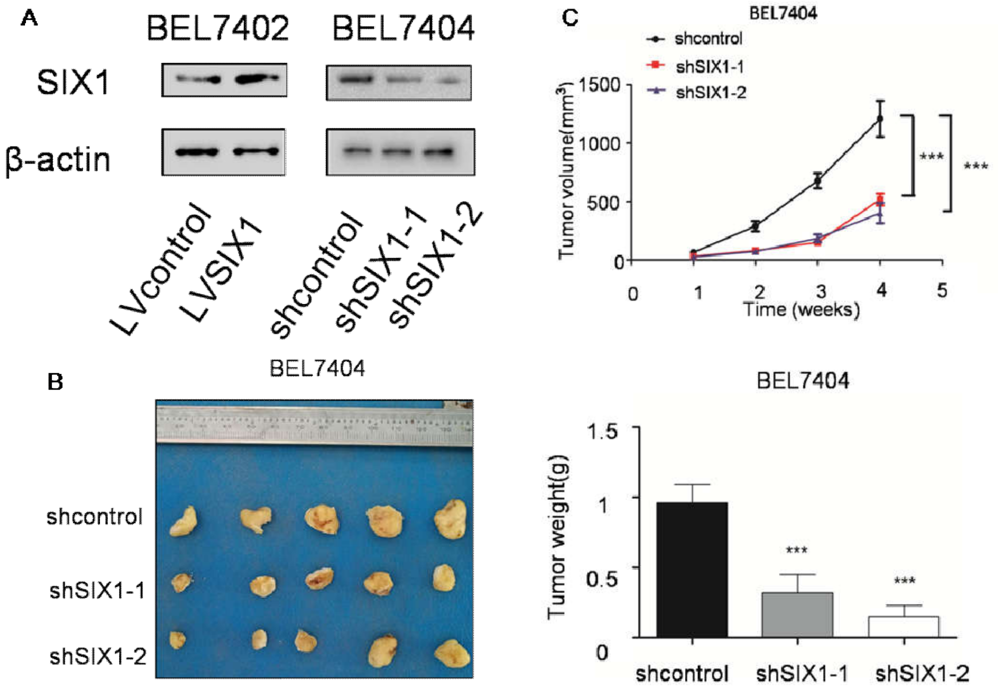

Supplementary Figure S1

A The efficiency of SIX1 manipulation using lentivirus was confirmed by western blot. B Tumors weights of nude mice were measured after four weeks at the experimental endpoint. C Plot of tumor volumes in nude mice measured every week (The data from B were analyzed by one-way ANOVA, and data from C were analyzed by two-way ANOVA. *$p < 0.05$, **$p < 0.01$, ***$p < 0.001$).

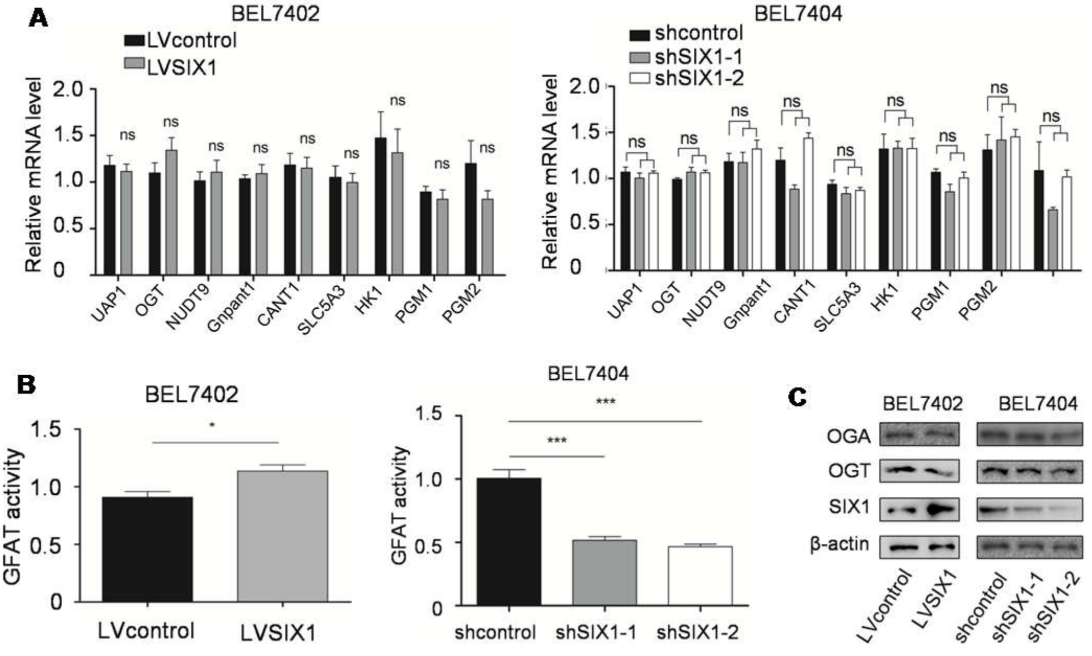

Supplementary Figure S2

A The mRNA levels of HBP related genes (UAP1, OGT, NUDT9, Gnpant1, CANT1, SLC5A3, HK1, PGM1, PGM2) were analyzed with qPCR in cells with SIX1 overexpression or knockdown. B GFAT activities were measured using GDH method in cells with SIX1 overexpression or knockdown. C OGT and OGA protein levels were analyzed by western blot in cells with SIX1 overexpression or knockdown. (The data were analyzed by Student's t-test (BEL7402) and one-way ANOVA (BEL7404). *$p < 0.05$, **$p < 0.01$, ***$p < 0.001$).

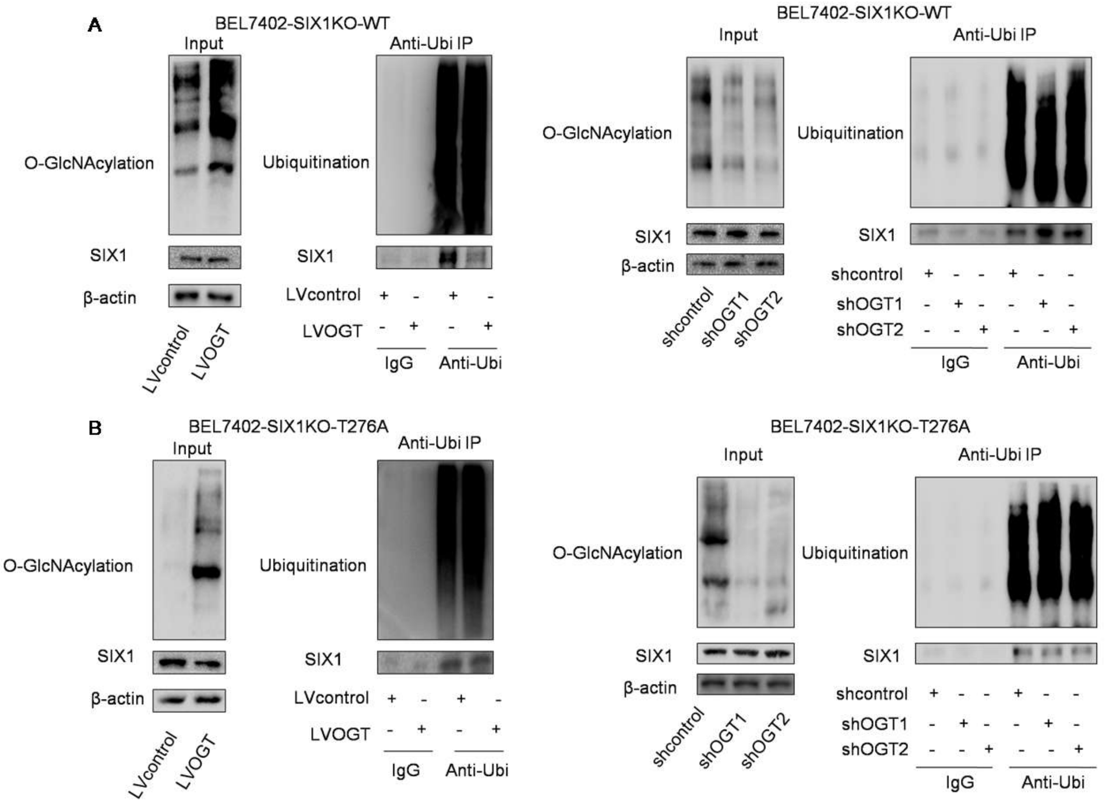

Supplementary Figure S3

The ubiquitination level of WT and T276A SIX1 protein in OGT overexpression and knockdown groups. BEL7402-SIX1KO cells were transfected with WT or mutant SIX1 plasmids and transduced with OGT overexpression or knockdown lentivirus.

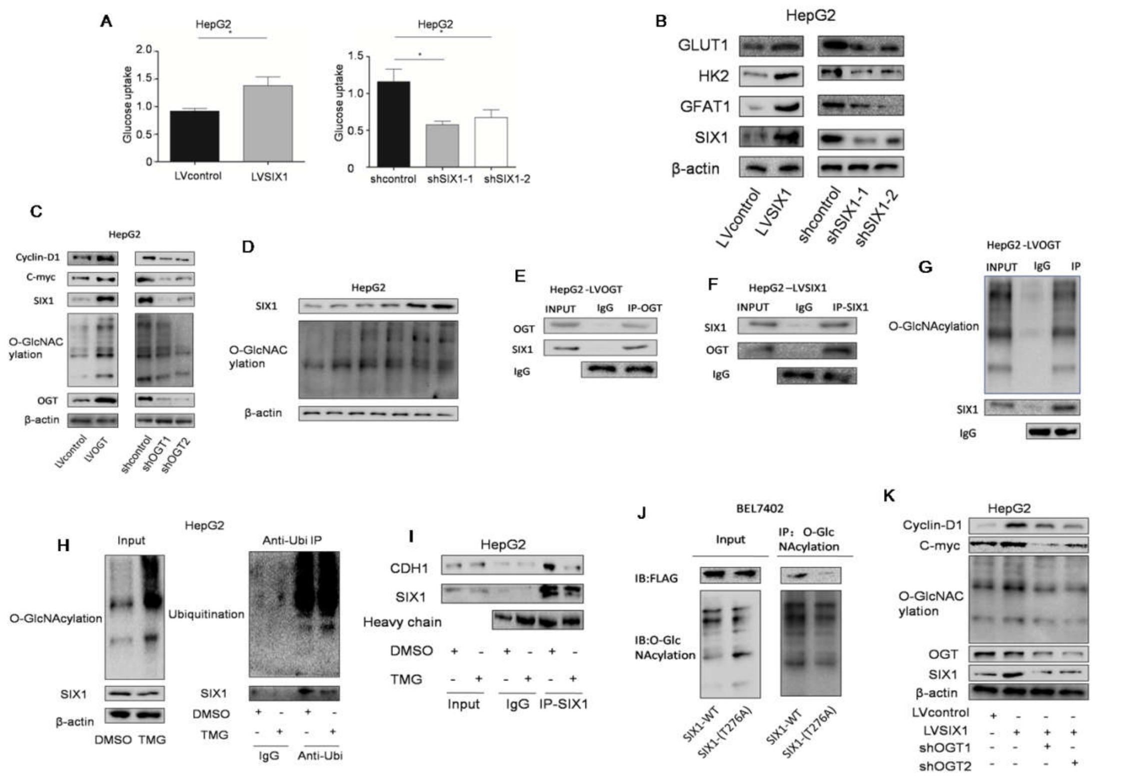

Supplementary Figure S4 The relationship between SIX1 and O-GlcNAcylation were confirmed using HepG2 cells. HepG2 cells were transduced with relevant SIX1 or OGT lentivirus.

A Glucose uptake levels were determined in HepG2 with different SIX1 expressions. B GLUT1, HK2, and GFAT1 expressions were confirmed using western blot. C SIX1 and its direct target genes were analyzed in HepG2 cells with different OGT expressions. D SIX1 expression in HepG2 cells treated with an OGA inhibitor (TMG) at the indicated times. E Immunoprecipitation with anti-OGT antibody followed by western blotting with anti-SIX1 antibody. F Immunoprecipitation with anti-SIX1 antibody followed by western blotting with anti-OGT antibody. G O-GlcNAcylation modified protein immunoprecipitated from cell extracts were analyzed by immunoblotting for anti-SIX1. H Ubiquitination of SIX1 was examined in HepG2 cells after OGA inhibitor treatment. MG132 and NEM were used to inhibit proteasome and deubiquitination, respectively. I CDH1 was detected in purified SIX1 Immunoprecipitation samples in HepG2 cells treated with DMSO or TMG. J The O-GlcNAcylation of Thr276 in SIX1 was analyzed by western blot in HepG2 cells. K SIX1 downstream factor protein levels and quantitative analysis in HepG2 treated as indicated were examined by western blot. (The data were analyzed by Student's t-test (BEL7402) and one-way ANOVA (BEL7404). *$p < 0.05$, **$p < 0.01$, ***$p < 0.001$).

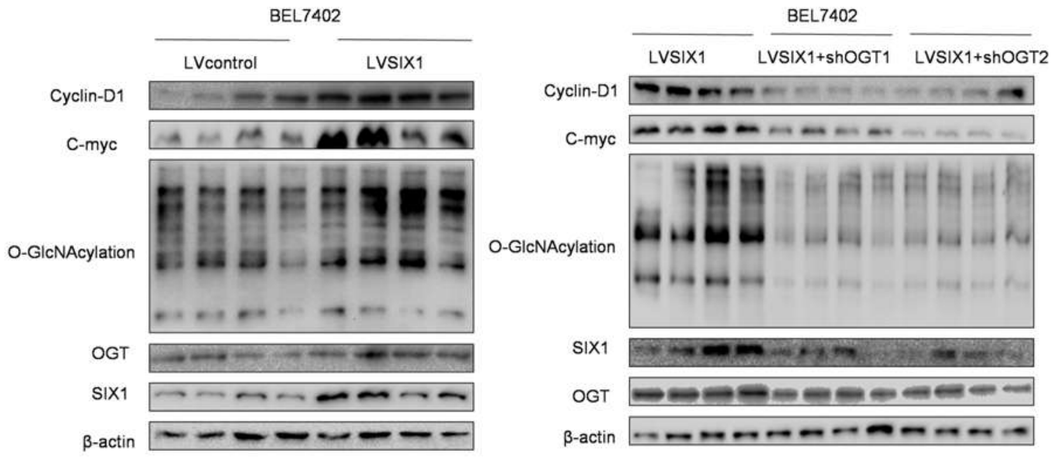

Supplementary Figure S5

Western blotting of the indicated factors in the tumors from the nude mice.

Supplementary Table 1

Primers for qPCR

Name	5'-3'
Glut1-qPCR-F	AAGAAGCTGACGGGTCGCCTCATGC
Glut1-qPCR-R	TGAGAGGGACCAGAGCGTGGTG
Nudt9-qPCR-F	GAAAAGTTGCACAAACTCTTCAG
Nudt9-qPCR-R	TCATTGATGTCCACCCATTTCAC
Gucy1a3-qPCR-F	GAAGCACAGCCCCGAGGTGTG
Gucy1a3-qPCR-R	ACTGACAAACAGAAGCCACTATC
Cant1-qPCR-F	GGCACCGTGGAGAAAGGCTTC
Cant1-qPCR-R	GACACCCAGTTCTCGTGGTCCAC
Gfpt1-qPCR-F	CTGATCACATTCCTATACTCTACAG
Gfpt1-qPCR-R	GTTCTATGACAGCACTTGCATC
Gnpant1-qPCR-F	CAGATTGTTGCTACGGCAACTC
Gnpant1-qPCR-R	GACATTCAAGGGTAATCTTGTAAC
Uap1-qPCR-F	GCAGTTCTTCTTCTAGCTGGTG
Uap1-qPCR-R	ATTCCATTGTTCTGCCACTGGTC
OGT-qPCR-F	GGCAGTACAAGCTTACGTCTCTG
OGT-qPCR-R	GTGCATTGAAAACACAGCCAAG
SLC5A3-qPCR-F	CATCAGGGATCTATACTCTTCAG
SLC5A3-qPCR-R	ACTAGGGACAGCCACTGGTATACAC
GAPDH-qPCR-F	ATCATCCCTGCCTCTACTGG
GAPDH-qPCR-R	GTCAGGTCCACCACTGACAC
Hk1-qPCR-F	AGTGGAAGGAGCAGATGTGGTC
Hk1-qPCR-R	TGTAGCAAGCATTGGTGCCAGTG
Hk2-qPCR-F	CTAAACTAGACGAGAGTTTCCTG
Hk2-qPCR-R	CTCACAGTTGTGGTCATCATAAC
SIX1-qPCR-F	CGCGCACAATCCCTACCCATCGCC
SIX1-qPCR-R	CTTCCAGAGGAGAGAGTTGGTTCTG
Tubulin-qPCR-F	CCAAGCTGGAGTTCTCTA
Tubulin-qPCR-R	CAATCAGAGTGCTCCAGG
Pgm1-qPCR-F	CCAAATCAGCAAGACAATTGAAG
Pgm1-qPCR-R	GTCGGTTTGGCCCAGAAAGTAGTTC
Pgm2-qPCR-F	CCTCTTTTCTGATATAACGCCAAC
Pgm2-qPCR-R	CCCTTTATCGTGAGGAGAAATG

Supplementary Table S2

1. Vector information：

Vector：GV141

Component order：CMV-MCS-3FLAG-SV40-Neomycin

Clone Site : XhoI / KpnI

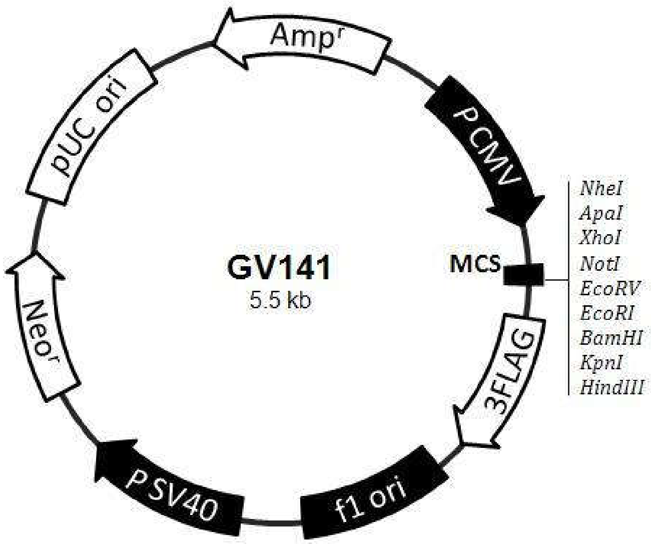

2. Primer in acquisition of target gene fragments and sequencing results

①SIX1 (wild type)

ID	sequence
SIX1 (wild type)-P1	ACGGGCCCTCTAGACTCGAGCGCCACCATGTCGATGCTGCCGTCGTTTGGC
SIX1 (wild type)-P2	AGTCACTTAAGCTTGGTACCGAGGACCCCAAGTCCACCAGACTGG

TGCTTACTGGCTTATCGAAATTAATACGACTCACTATAGGGAGACCCAAGCTGGCTAGC

GTTTAAACGGGCCCTCTAGACTCGAGCGCCACCATGTCGATGCTGCCGTCGTTTGGCTT

TACGCAGGAGCAAGTGGCGTGCGTGTGCGAGGTTCTGCAGCAAGGCGGAAACCTGGA

GCGCCTGGGCAGGTTCCTGTGGTCACTGCCCGCCTGCGACCACCTGCACAAGAACGA

GAGCGTACTCAAGGCCAAGGCGGTGGTCGCCTTCCACCGCGGCAACTTCCGTGAGCT

CTACAAGATCCTGGAGAGCCACCAGTTCTCGCCTCACAACCACCCCAAACTGCAGCAA

CTGTGGCTGAAGGCGCATTACGTGGAGGCCGAGAAGCTGCGCGGCCGACCCCTGGGC

GCCGTGGGCAAATATCGGGTGCGCCGAAAATTTCCACTGCCGCGCACCATCTGGGACG

GCGAGGAGACCAGCTACTGCTTCAAGGAGAAGTCGAGGGGTGTCCTGCGGGAGTGGT

ACGCGCACAATCCCTACCCATCGCCGCGTGAGAAGCGGGAGCTGGCCGAGGCCACCG

GCCTCACCACCACCCAGGTCAGCAACTGGTTTAAGAACCGGAGGCAAAGAGACCGGG

CCGCGGAGGCCAAGGAAAGGGAGAACACCGAAAACAATAACTCCTCCTCCAACAAGC

AGAACCAACTCTCTCCTCTGGAAGGGGGCAAGCCGCTCATGTCCAGCTCAGAAGAGG

AATTCTCACCTCCCCAAAGTCCAGACCAGAACTCGGTCCTTCTGCTGCAGGGCAATAT

GGGCCACGCCAGGAGCTCAAACTATTCTCTCCCGGGCTTAACAGCCTCGCAGCCCAGT

CACGGCCTGCAGACCCACCAGCATCAGCTCCAAGACTCTCTGCTCGGCCCCCTCACCT

CCAGTCTGGTGGACTTGGGGTCCTCGGTACCAAGCTTAAGTGACTACAAGGATGACGA

TGACAAGGATTACAAAGACGACGATGATAAGGACTATAAGGATGATGACGACAAATCT

AGATAGTTAAACCGCTGATCAGCCTCG

②SIX1 (S225A)

ID	sequence
SIX1(S225A)-p1	ACGGGCCCTCTAGACTCGAGCGCCACCATGTCGATGCTGCCGTCGTTTGG
SIX1(S225A)-p2	AGTCACTTAAGCTTGGTACCGAGGACCCCAAGTCCACCAGACTGG

SIX1(S225A)-p3	CACCTCCCCAAGCTCCAGACCAGAACTCGGTCCTTC
SIX1(S225A)-p4	CTGGTCTGGAGCTTGGGGAGGTGAGAATTCCTCTTC

ACTGGCTTATCGAAATTAATACGACTCACTATAGGGAGACCCAAGCTGGCTAGCGTTTA

AACGGGCCCTCTAGACTCGAGCGCCACCATGTCGATGCTGCCGTCGTTTGGCTTTACG

CAGGAGCAAGTGGCGTGCGTGTGCGAGGTTCTGCAGCAAGGCGGAAACCTGGAGCG

CCTGGGCAGGTTCCTGTGGTCACTGCCCGCCTGCGACCACCTGCACAAGAACGAGAG

CGTACTCAAGGCCAAGGCGGTGGTCGCCTTCCACCGCGGCAACTTCCGTGAGCTCTAC

AAGATCCTGGAGAGCCACCAGTTCTCGCCTCACAACCACCCCAAACTGCAGCAACTG

TGGCTGAAGGCGCATTACGTGGAGGCCGAGAAGCTGCGCGGCCGACCCCTGGGCGCC

GTGGGCAAATATCGGGTGCGCCGAAAATTTCCACTGCCGCGCACCATCTGGGACGGCG

AGGAGACCAGCTACTGCTTCAAGGAGAAGTCGAGGGGTGTCCTGCGGGAGTGGTACG

CGCACAATCCCTACCCATCGCCGCGTGAGAAGCGGGAGCTGGCCGAGGCCACCGGCC

TCACCACCACCCAGGTCAGCAACTGGTTTAAGAACCGGAGGCAAAGAGACCGGGCCG

CGGAGGCCAAGGAAAGGGAGAACACCGAAAACAATAACTCCTCCTCCAACAAGCAG

AACCAACTCTCTCCTCTGGAAGGGGGCAAGCCGCTCATGTCCAGCTCAGAAGAGGAA

TTCTCACCTCCCCAAGCTCCAGACCAGAACTCGGTCCTTCTGCTGCAGGGCAATATGG

GCCACGCCAGGAGCTCAAACTATTCTCTCCCGGGCTTAACAGCCTCGCAGCCCAGTCA

CGGCCTGCAGACCCACCAGCATCAGCTCCAAGACTCTCTGCTCGGCCCCCTCACCTCC

AGTCTGGTGGACTTGGGGTCCTCGGTACCAAGCTTAAGTGACTACAAGGATGACGATG

ACAAGGATTACAAAGACGACGATGATAAGGACTATAAGGATGATGACGACAAATCTAG

ATAGTTAAACCGCTGATCAGCCTCGACTGTGCCTTCTA

③SIX1 (T252A)

ID	sequence
SIX1 (T252A)-p1	ACGGGCCCTCTAGACTCGAGCGCCACCATGTCGATGCTGCCGTCGTTTG
SIX1 (T252A)-p2	GCGAGGCTGCTAAGCCCGGGAGAGAATAGTTTG
SIX1 (T252A)-p3	CCCGGGCTTAGCAGCCTCGCAGCCCAGTCAC
SIX1 (T252A)-p4	AGTCACTTAAGCTTGGTACCGAGGACCCCAAGTCCACCAGACTGG

AGCTCTCTGGCTAACTAGAGAACCCACTGCTTACTGGCTTATCGAAATTAATACGACTC

ACTATAGGGAGACCCAAGCTGGCTAGCGTTTAAACGGGCCCTCTAGACTCGAGCGCCA
CCATGTCGATGCTGCCGTCGTTTGGCTTTACGCAGGAGCAAGTGGCGTGCGTGTGCGA
GGTTCTGCAGCAAGGCGGAAACCTGGAGCGCCTGGGCAGGTTCCTGTGGTCACTGCC
CGCCTGCGACCACCTGCACAAGAACGAGAGCGTACTCAAGGCCAAGGCGGTGGTCGC
CTTCCACCGCGGCAACTTCCGTGAGCTCTACAAGATCCTGGAGAGCCACCAGTTCTCG
CCTCACAACCACCCCAAACTGCAGCAACTGTGGCTGAAGGCGCATTACGTGGAGGCC
GAGAAGCTGCGCGGCCGACCCCTGGGCGCCGTGGGCAAATATCGGGTGCGCCGAAAA
TTTCCACTGCCGCGCACCATCTGGGACGGCGAGGAGACCAGCTACTGCTTCAAGGAG
AAGTCGAGGGGTGTCCTGCGGGAGTGGTACGCGCACAATCCCTACCCATCGCCGCGTG
AGAAGCGGGAGCTGGCCGAGGCCACCGGCCTCACCACCACCCAGGTCAGCAACTGGT
TTAAGAACCGGAGGCAAAGAGACCGGGCCGCGGAGGCCAAGGAAAGGGAGAACACC
GAAAACAATAACTCCTCCTCCAACAAGCAGAACCAACTCTCTCCTCTGGAAGGGGGC
AAGCCGCTCATGTCCAGCTCAGAAGAGGAATTCTCACCTCCCCAAAGTCCAGACCAG
AACTCGGTCCTTCTGCTGCAGGGCAATATGGGCCACGCCAGGAGCTCAAACTATTCTC
TCCCGGGCTTAGCAGCCTCGCAGCCCAGTCACGGCCTGCAGACCCACCAGCATCAGCT
CCAAGACTCTCTGCTCGGCCCCCTCACCTCCAGTCTGGTGGACTTGGGGTCCTCGGTA
CCAAGCTTAAGTGACTACAAGGATGACGATGACAAGGATTACAAAGACGACGATGATA
AGGACTATAAGGATGATGACGACAAATCTAGATAGTTAAACCGCTGATCAGCCTCGACT
GTGCCTT

④SIX1 (T276A)

ID	sequence
SIX1 (T276A)-P1	ACGGGCCCTCTAGACTCGAGCGCCACCATGTCGATGCTGCCGTCGTTTG
SIX1 (T276A)-P2	AGTCACTTAAGCTTGGTACCGAGGACCCCAAGTCCACCAGACTGGAGGCGAGGGGGCCGAGCAGAGAGTC

AGAACCCACTGCTTACTGGCTTATCGAAATTAATACGACTCACTATAGGGAGACCCAAG
CTGGCTAGCGTTTAAACGGGCCCTCTAGACTCGAGCGCCACCATGTCGATGCTGCCGT
CGTTTGGCTTTACGCAGGAGCAAGTGGCGTGCGTGTGCGAGGTTCTGCAGCAAGGCG

GAAACCTGGAGCGCCTGGGCAGGTTCCTGTGGTCACTGCCCGCCTGCGACCACCTGC

ACAAGAACGAGAGCGTACTCAAGGCCAAGGCGGTGGTCGCCTTCCACCGCGGCAACT

TCCGTGAGCTCTACAAGATCCTGGAGAGCCACCAGTTCTCGCCTCACAACCACCCCAA

ACTGCAGCAACTGTGGCTGAAGGCGCATTACGTGGAGGCCGAGAAGCTGCGCGGCCG

ACCCCTGGGCGCCGTGGGCAAATATCGGGTGCGCCGAAAATTTCCACTGCCGCGCACC

ATCTGGGACGGCGAGGAGACCAGCTACTGCTTCAAGGAGAAGTCGAGGGGTGTCCTG

CGGGAGTGGTACGCGCACAATCCCTACCCATCGCCGCGTGAGAAGCGGGAGCTGGCC

GAGGCCACCGGCCTCACCACCACCCAGGTCAGCAACTGGTTTAAGAACCGGAGGCAA

AGAGACCGGGCCGCGGAGGCCAAGGAAAGGGAGAACACCGAAAACAATAACTCCTC

CTCCAACAAGCAGAACCAACTCTCTCCTCTGGAAGGGGGCAAGCCGCTCATGTCCAG

CTCAGAAGAGGAATTCTCACCTCCCCAAAGTCCAGACCAGAACTCGGTCCTTCTGCTG

CAGGGCAATATGGGCCACGCCAGGAGCTCAAACTATTCTCTCCCGGGCTTAACAGCCT

CGCAGCCCAGTCACGGCCTGCAGACCCACCAGCATCAGCTCCAAGACTCTCTGCTCGG

CCCCCTCGCCTCCAGTCTGGTGGACTTGGGGTCCTCGGTACCAAGCTTAAGTGACTAC

AAGGATGACGATGACAAGGATTACAAAGACGACGATGATAAGGACTATAAGGATGATG

ACGACAAATCTAGATAGTTAAACCGCTGATCAGCCTCGACTGTGCCTTCTAGTTGCCAG

Supplementary Table S3

Mass spectrum results of potential OGT binding targets in BEL7402-LVOGT cell

Mass spectrum analysis results						
**#**	**Symbol**	**Matched Term**	**Synonym(s)**	**Entrez Gene Name**	**Location**	**Type(s)**
1	ABO	Q99480	A3GALNT,A3GALT1,ABO blood group (transferase A, alpha 1-3-N-acetylgalactosaminyltransferase, transferase B, alpha 1-3-galactosyltransferase),ABO blood group (transferase A, α 1-3-N-acetylgalactosaminyltransferase, transferase B, α 1-3-galactosyltransferase),ABO, alpha 1-3-N-acetylgalactosaminyltransferase and alpha 1-3-galactosyltransferase,ABO, α 1-3-N-acetylgalactosaminyltransferase and α 1-3-galactosyltransferase,Abo2,Abo3, Abol1,Gbgt1,GTB,histo-blood group ABO system transferase 2,histo-blood group ABO system transferase 3,LOC687450,NAGAT,NAGAT 2	ABO, alpha 1-3-N-acetylgalactosaminyltransferase and alpha 1-3-galactosyltransferase	Plasma Membrane	enzyme
2	ACTB	B4DW52	A-X actin,actin,Actin beta,Actin β,actin, beta,actin, β,Actx,beta-actin,BRWS1,E430023M04Rik,Melanoma x actin,PS1TP5BP1,βCa,β-actin	actin beta	Cytoplasm	other
3	ACTL6A	O96019	2810432C06Rik,Actin-like 6a,ACTL6,AI851094,ARP4,ARPN-BETA, ARPN-β,Baf53,BAF53A,C79802,INO80K,RGD1307747	actin like 6A	Nucleus	other
4	ACTN4	O43707	ACTININ 4,actinin alpha 4,actinin α 4,actinin, alpha 4,Alpha actinin 4,C77391,FSGS,FSGS1,NON-MUSCLE alpha-ACTININ,NON-MUSCLE α-ACTININ,α-actinin-4	actinin alpha 4	Cytoplasm	transcription regulator
5	ADD3	Q53FL4	35H,ADDL,adducin 3,adducin 3 (gamma),adducin 3 (γ),Adducin-gamma,Adducin-γ,AI4632	adducin 3	Cytoplasm	other

			85,CPSQ3,gamma ADDUCIN,R75380,γ ADDUCIN			
6	AFG3L2	D3DUJ0	2310036I02RIK,AFG3 like matrix AAA peptidase subunit 2,AFG3-like AAA ATPase 2,AW260507,Emv66,par,SCA28,SPAX5	AFG3 like matrix AAA peptidase subunit 2	Cytoplasm	peptidase
7	AGK	E9PG39	2610037M15Rik,6720408I04RIK,acylglycerol kinase,AI465370,CATC5,CTRCT38,FLJ10842,MTDPS10,MULK,RGD1562046	acylglycerol kinase	Cytoplasm	kinase
8	AGRN	O00468	AGR,AGRIN,CMS8,CMSPPD,LOC100503504,Neural agrin,nmf380	agrin	Plasma Membrane	other
9	ANP32E	E9PPH5	2810018A15Rik,acidic nuclear phosphoprotein 32 family member E,AI047746,AI326868,CPD1,LANP-L,mLANP-L	acidic nuclear phosphoprotein 32 family member E	Nucleus	other
10	AP2A2	O94973	2410074K14Rik,Adaptin alpha,Adaptin α,adaptor related protein complex 2 subunit alpha 2,adaptor related protein complex 2 subunit α 2,adaptor-related protein complex 2, alpha 2 subunit,adaptor-related protein complex 2, α 2 subunit,ADTAB,AF006990,Alpha-adaptin C,AP-2 alpha C subunit,AP-2 α C subunit,AP2,Ap2 alpha,Ap2 alpha C,Ap2 α,Ap2 α C,AW146353,C78001,CLAPA2,HIP-9,HYPJ,L25,LOC101927462,α-adaptin C	adaptor related protein complex 2 subunit alpha 2	Cytoplasm	transporter
11	AQR	A8K6F0	aquarius,aquarius intron-binding spliceosomal factor,AW495846,fSAP164,IBP160,mKIAA0560	aquarius intron-binding spliceosomal factor	Nucleus	other
12	ARID1A	H0Y488	1110030E03Rik,AT rich interactive domain 1A (SWI-like),AT-rich interaction domain 1A,B120,BAF250,BAF250A,BM029,C1orf4,CSS2,ELD,hELD,hOSA1,HSP16.2,M	AT-rich interaction domain 1A	Nucleus	transcription regulator

			RD14,OSA1,P270,SMARCF1			
13	ARMCX3	A8K5B0	1200004E24Rik,AI450003,ALEX3,armadillo repeat containing X-linked 3,armadillo repeat containing, X-linked 3,BM017,dJ545K15.2,GASP6	armadillo repeat containing X-linked 3	Cytoplasm	other
14	ATP1A1	P05023	ATP1A1 isoform 1,Atpa-1,ATPase Na+/K+ transporting subunit alpha 1,ATPase Na+/K+ transporting subunit α 1,ATPase, Na+/K+ transporting, alpha 1 polypeptide,ATPase, Na+/K+ transporting, α 1 polypeptide,BC010319,CMT2DD,F1 ATPase alpha,F1 ATPase α,Lactate dehydrogenase 1, a chain,Na K α 1,Na,K-ATPase α subunit 1,Na/K atpase alpha 1,Na/K atpase alpha 3,Na/K atpase α 1,Na/K atpase α 3,NKA alpha 1,NKA α 1,Nkaa1b	ATPase Na+/K+ transporting subunit alpha 1	Plasma Membrane	transporter
15	ATP2A2	P16615	9530097L16RIK,ATP2B,ATPase sarcoplasmic/endoplasmic reticulum Ca2+ transporting 2,ATPase, Ca++ transporting, cardiac muscle, slow twitch 2,Ca-transporting atpase sarcoplasmic reticulum type,D5Wsu150e,DAR,DD,mKIAA4195, Sarcoplasmic reticulum ca2+ atpase,SERCA2,Serca2a,Serca2a/b,SercaII	ATPase sarcoplasmic/endoplasmic reticulum Ca2+ transporting 2	Cytoplasm	transporter
16	ATP5F1C	B4DL14	1700094F02Rik,ATP synthase F1 subunit gamma,ATP synthase F1 subunit γ,ATP synthase, H+ transporting, mitochondrial F1 complex, gamma polypeptide 1,ATP synthase, H+ transporting, mitochondrial F1 complex, γ polypeptide 1,ATP5C,ATP5C1,ATP5CL1,F1 γ,γ subunit f1f0 atpase	ATP synthase F1 subunit gamma	Cytoplasm	transporter
17	AURKB	J3QR41	AIK2,AIM-1,AIRK2,AL022959,ARK-2,AurB,aurkb-sv1,aurkb-sv2,AURORA B,aurora kinase B,IPL1,PPP1R48,Serine/threonine kinase 5,STK-1,STK12,STK5	aurora kinase B	Nucleus	kinase

18	BAZ1B	Q9UIG0	bromodomain adjacent to zinc finger domain 1B,bromodomain adjacent to zinc finger domain, 1B,C87820,WBSCR10,WBSCR9,WSTF	bromodomain adjacent to zinc finger domain 1B	Nucleus	transcription regulator
19	BMS1	Q14692	AA408648,ACC,AU020092,AU043373, BB007109,BC030906,BMS1 ribosome biogenesis factor,BMS1, ribosome biogenesis factor,BMS1L,KIAA0187,mKIAA0187	BMS1, ribosome biogenesis factor	Nucleus	other
20	CALR	Q53G71	Calbindin-57kd,Calregulin,calreticulin, CALRETICULIN isoform 1,cC1qR,CRT,HEL-S-99n,RO,SSA	calreticulin	Cytoplasm	transcription regulator
21	CAPZA1	A8K0T9	CAPPA1,capping actin protein of muscle Z-line subunit alpha 1,capping actin protein of muscle Z-line subunit α 1,capping protein (actin filament) muscle Z-line, alpha 1,capping protein (actin filament) muscle Z-line, α 1,CAPZ,capZ alpha-1,capZ α-1,CAZ1,LOC684096	capping actin protein of muscle Z-line subunit alpha 1	Cytoplasm	other
22	CCDC47	Q96A33	2610204L23RIK,asp4,C88307,calumin, coiled-coil domain containing 47,D13Mgi22,GK001,MSTP041,RGD1308813	coiled-coil domain containing 47	Extracellular Space	other
23	CCT3	B4DUR8	AL024092,CCT-gamma,CCT-γ,Cct1 γ,CCTG,chaperonin containing TCP1 subunit 3,chaperonin containing Tcp1, subunit 3 (gamma),chaperonin containing Tcp1, subunit 3 (γ),Chaperonin subunit 3 (gamma),Chaperonin subunit 3 (γ),Matricin,PIG48,TCP-1-gamma,TCP-1-γ,Tcp1-rs3,TriC-P5,TRIC5	chaperonin containing TCP1 subunit 3	Cytoplasm	other
24	CCT4	B7Z9L0	2610204B21RIK,A45,C78323,CCT-DELTA,CCT-δ,Cctd,chaperonin containing TCP1 subunit 4,chaperonin containing Tcp1, subunit 4 (delta),chaperonin containing Tcp1, subunit 4 (δ),SRB,T complex protein 1, delta subunit,T complex protein 1, δ subunit,TCP1 delta,TCP1 δ	chaperonin containing TCP1 subunit 4	Cytoplasm	other

25	CD44	H0YD13	216062 AT,AU023126,AW121933,AW146109,CD44 (containing exon 5),CD44 (soluble),CD44 Antigen,CD44 molecule (Indian blood group),CD44 STANDARD FROM,CD44A,Cd44e,Cd44i,CDW44,CSPG8,ECMR-III,Epican,HCELL,HERMES,Hermes antigen,HUTCH-I,IN,LHR,Ly-24,MC56,MDU2,MDU3,METAA,MIC4,NKT.44,Pgp-1,RHAMM	CD44 molecule (Indian blood group)	Plasma Membrane	other
26	CDK12	Q9NYV4	1810022J16RIK,AI646528,CRK7,CRKR,CRKRS,cyclin-dependent kinase 12,D11Ertd752e,LOC100502717,Pksc	cyclin dependent kinase 12	Nucleus	kinase
27	CES4A	Q5XG92	carboxylesterase 4A,CES6,CES8,RGD1307418	carboxylesterase 4A	Extracellular Space	enzyme
28	CHD2	A8K9Y5	2810013C04RIK,2810040A01Rik,5630401D06RIK,AI851092,BC029703,chromodomain helicase DNA binding protein 2,EEOC,LOC283680	chromodomain helicase DNA binding protein 2	Nucleus	enzyme
29	CHERP	Q8IWX8	5730408I11Rik,calcium homeostasis endoplasmic reticulum protein,D8Wsu96e,DAN16,ERPROT213-21,SCAF6,SRA1	calcium homeostasis endoplasmic reticulum protein	Cytoplasm	other
30	CKAP5	Q14008	3110043H24Rik,4930432B04RIK,CHTOG,cytoskeleton associated protein 5,D730027C18RIK,mKIAA0097,MSPS,T25636,TOG,TOGP	cytoskeleton associated protein 5	Nucleus	transcription regulator
31	CLDN2	P57739	AL022813,Claudin-2,RGD1560247	claudin 2	Plasma Membrane	other
32	COPA	P53621	AILJK,Alpha-coat,Alpha-COP,AU040324,coatomer protein complex subunit alpha,coatomer protein complex subunit α,HEP-COP,xenin,α-coat,α-COP	coatomer protein complex subunit alpha	Cytoplasm	transporter

33	CORO1C	B7Z9V0	AL022675,AW455561,AW548837,coronin 1C,coronin, actin binding protein 1C,CRN2,HCRNN4,RGD1564490	coronin 1C	Cytoplasm	other
34	CSTF1	B4DDG3	1700057K18Rik,AI788832,CF1,cleavage stimulation factor subunit 1,cleavage stimulation factor, 3' pre-RNA, subunit 1,CstF-50,CstFp50	cleavage stimulation factor subunit 1	Nucleus	other
35	CTNND1	O60716	AA409437,AU019353,BCDS2,CAS,catenin (cadherin associated protein), delta 1,catenin (cadherin associated protein), δ 1,catenin delta 1,catenin δ 1,CATNS,CTNN delta,CTNN delta1,CTNN δ,CTNN δ1,CTNND,mKIAA0384,P120,p120 Catenin,p120(CAS),p120(CTN),P120CAS,P120CTN,Pp120	catenin delta 1	Nucleus	other
36	DBN1	Q16643	D0S117E,drebrin 1,Drebrin E,DREBRIN E2	drebrin 1	Cytoplasm	other
37	DDX10	Q86VR6	4632415A01Rik,AI646054,Dbp4,DEAD (Asp-Glu-Ala-Asp) box polypeptide 10,DEAD-box helicase 10,HRH-J8	DEAD-box helicase 10	Nucleus	enzyme
38	DDX18	Q4ZG72	2310005B10Rik,DEAD (Asp-Glu-Ala-Asp) box polypeptide 18,DEAD-box helicase 18,Has1,MrDb	DEAD-box helicase 18	Nucleus	enzyme
39	DDX24	G3V529	1700055J08Rik,2510027P10Rik,AI649272,DEAD (Asp-Glu-Ala-Asp) box polypeptide 24,DEAD-box helicase 24	DEAD-box helicase 24	Nucleus	enzyme
40	DDX39B	Q13838	0610030D10Rik,AI428441,BAT1,Bat1a,D17H6S81E,D17H6S81E-1,D6S81E,D6S81Eh,DEAD (Asp-Glu-Ala-Asp) box polypeptide 39B,Dead Box RNA Helicase p47,DExD-box helicase 39B,P47,UAP56	DExD-box helicase 39B	Nucleus	enzyme
41	DDX41	B3KRK2	2900024F02Rik,AA958953,ABS,AI324246,DEAD (Asp-Glu-Ala-Asp) box polypeptide 41,DEAD-box helicase 41,MPLPF	DEAD-box helicase 41	Nucleus	enzyme
42	DDX55	B4DVE4	2810021H22Rik,DEAD (Asp-Glu-Ala-Asp) box polypeptide 55,DEAD-box helicase 55,mKIAA1595	DEAD-box helicase 55	Nucleus	enzyme
43	DECR1	Q16698	1200012F07Rik,2,4 Dienoyl Reductase,2,4-Dienoyl coa reductase,2,4-dienoyl CoA reductase 1, mitochondrial,2,4-dienoyl-CoA	2,4-dienoyl-CoA reductase 1	Cytoplasm	enzyme

			reductase 1,DECR,NADPH,SDR18C1			
44	DHCR24	Q3LIE7	2310076D10Rik,24-Dehydrocholesterol Reductase,24-Dhcr,3-beta--hydroxysterol delta-24-reductase,3-β--hydroxysterol δ-24-reductase,5830417J06Rik,DCE,KIAA0018,mKIAA0018,Nbla03646,SELADIN1	24-dehydrocholesterol reductase	Cytoplasm	enzyme
45	DHX16	Q9P186	2410006N22Rik,DBP2,DDX16,DEAH (Asp-Glu-Ala-His) box polypeptide 16,DEAH-box helicase 16,KIAA0577,mKIAA0577,PRO2014,Prp2,PRP8,PRPF2	DEAH-box helicase 16	Nucleus	enzyme
46	DHX36	E7EWK3	2810407E23Rik,AI452301,AU022184,DDX36,DEAH (Asp-Glu-Ala-His) box polypeptide 36,DEAH-box helicase 36,G4R1,mKIAA1488,MLEL1,RHAU	DEAH-box helicase 36	Cytoplasm	enzyme
47	DKC1	O60832	BC068171,CBF5,DKC,DKCX,dyskeratosis congenita 1, dyskerin,dyskerin pseudouridine synthase 1,NAP57,NOLA4,Weakly similar to tyrosine-prtein kinase jak3,XAP101	dyskerin pseudouridine synthase 1	Nucleus	enzyme
48	DNAJB11	Q9UBS4	1810031F23Rik,ABBP-2,AL024055,DJ9,DnaJ heat shock protein family (Hsp40) member B11,EDJ,ERdj3,ERj3,ERj3p,LRRGT00084,PRO1080,UNQ537	DnaJ heat shock protein family (Hsp40) member B11	Cytoplasm	other
49	DPYSL3	H0YBT4	CRMP-4,DIHYDROPYRIMIDINASE RELATED protein-3,dihydropyrimidinase-like 3,DRP-3,LCRMP,Tuc-4a,TUC-4b,TUC4,ULIP,ULIP-1	dihydropyrimidinase like 3	Cytoplasm	enzyme
50	EEF1B2	F8WF65	2810017J07Rik,EEF1B,EEF1B1,EF-1 β,EF1B,ELONGATION FACTOR 1 beta,ELONGATION FACTOR 1 β,eukaryotic translation elongation factor 1 beta 2,eukaryotic translation elongation factor 1 β 2	eukaryotic translation elongation factor 1 beta 2	Cytoplasm	translation regulator
51	EEF1D	P29692	1700026P12RIK,Eef1 δ,EF-1-delta,EF-1-δ,EF-1D,EF1B	eukaryotic translatio	Cytoplasm	translation

			beta,EF1B β,eukaryotic translation elongation factor 1 delta,eukaryotic translation elongation factor 1 delta (guanine nucleotide exchange protein),eukaryotic translation elongation factor 1 δ,eukaryotic translation elongation factor 1 δ (guanine nucleotide exchange protein),FP1047,Tef1 delta,Tef1 δ,UNQ601,δ Ef1	n elongation factor 1 delta		regulator
52	EIF2AK2	P19525	2310047A08Rik,4732414G15Rik,AI467567,AI747578,DAI,DOUBLE STRANDED RNA DEPENDENT protein KINASE,EIF2AK1,eukaryotic translation initiation factor 2-alpha kinase 2,eukaryotic translation initiation factor 2-α kinase 2,IFNPKR,p68 kinase,PKR,PPP1R83,PRKR,Tik	eukaryotic translation initiation factor 2 alpha kinase 2	Cytoplasm	kinase
53	EIF2S1	Q53XC0	0910001O23Rik,2410026C18Rik,EIF-2, EIF-2A,EIF-2alpha,eIF-2α,Eukaryotic Translation Initiation Factor 2 Alpha Subunit,eukaryotic translation initiation factor 2 subunit alpha,eukaryotic translation initiation factor 2 subunit α,Eukaryotic Translation Initiation Factor 2 α Subunit,eukaryotic translation initiation factor 2, subunit 1 alpha,eukaryotic translation initiation factor 2, subunit 1 α	eukaryotic translation initiation factor 2 subunit alpha	Cytoplasm	translation regulator
54	EIF2S3	P41091	AA409828,AA547477,AI314668,eIF-2gA,Eif-2gx,EIF2,Eif2 γ,EIF2G,EIF2gamma,Eif2s3x,eukaryotic translation initiation factor 2 subunit gamma,eukaryotic translation initiation factor 2 subunit γ,Eukaryotic translation initiation factor 2, subunit 3,eukaryotic translation initiation factor 2, subunit 3, structural gene X-linked,MEHMO,MRXSBRK,PP42	eukaryotic translation initiation factor 2 subunit gamma	Cytoplasm	translation regulator
55	EIF3F	B4DMT5	0610037M02Rik,AA409853,eIF3-p47,Eif3-ε,EIF3S5,eukaryotic translation initiation factor 3 subunit F,eukaryotic translation initiation factor 3, subunit	eukaryotic translation initiation	Cytoplasm	translation regulator

			F	factor 3 subunit F		
56	ENO1	P06733	0610008I15,AL022784,EG433182,Eno1b,ENO1L1,ENOLASE,ENOLASE 1,Enolase 1 alpha,Enolase 1 α,enolase 1, alpha non-neuron,enolase 1, α non-neuron,enolase 1B, retrotransposed,Enolase α,Gm5506,HEL-S-17,LOC100503183,MBP-1,MPB1,NNE,Phosphopyruvate hydratase,PPH,α enolase	enolase 1	Cytoplasm	enzyme
57	EPHB4	Q96L35	AI042935,b2b2412Clo,EPH receptor B4,Ephrin b4,HFASD,HTK,MDK2,MYK1,TYRO11	EPH receptor B4	Plasma Membrane	kinase
58	EPPK1	P58107	6230424I18Rik,EPIPL,EPIPL1,epiplakin 1,EPPK,Eppk1-ps1,LOC686567,PH0268 epidermal autoantigen 450K	epiplakin 1	Cytoplasm	other
59	ERAL1	O75616	2610524P08Rik,9130407C09Rik,AU019798,CEGA,ERA,Era (G-protein)-like 1 (E. coli),Era-like 12S mitochondrial rRNA chaperone 1,ERA-W,ERAL1A,ERAL1B,H-ERA,HERA-A,HERA-B,M-ERA,MERA-S,MERA-W,PRLTS6	Era like 12S mitochondrial rRNA chaperone 1	Cytoplasm	other
60	EZH2	F2YMM1	EHZ2,enhancer of zeste 2 polycomb repressive complex 2 subunit,ENX-1,Enx1h,EZH2b,KMT6,KMT6A,mKIAA4065,WVS,WVS2	enhancer of zeste 2 polycomb repressive complex 2 subunit	Nucleus	transcription regulator
61	EZR	Q6NUR7	AW146364,CVIL,CVL,Cytovillin2,EZRIN,HEL-S-105,p81,R75297,VIL2	ezrin	Plasma Membrane	other
62	FAM111B	B4E0T6	CANP,family with sequence similarity 111 member B,POIKTMP	family with sequence similarity 111 member B	Other	other
63	FAM120A	Q9NZB2	C9orf10,family with sequence similarity 120, member A,family with sequence similarity 120A,HBVPTPAP,LOC679485,OSSA,RGD1306643,Similar to protein C9orf10	family with sequence similarity 120A	Cytoplasm	other

64	FARSA	K7ER16	0610012A19Rik,CML33,FARSL,FARSLA, FRSA,PheHA,Phenylalanine-tRNA synthetase-like, alpha-subunit,Phenylalanine-tRNA synthetase-like, α-subunit,phenylalanyl-tRNA synthetase subunit alpha,phenylalanyl-tRNA synthetase subunit α,phenylalanyl-tRNA synthetase, alpha subunit,phenylalanyl-tRNA synthetase, α subunit,pheRS	phenylalanyl-tRNA synthetase subunit alpha	Cytoplasm	enzyme
65	FASN	P49327	A630082H08Rik,FAS,fatty acid synthase,Long chain fatty acyl-coa synthetase,NADP Dependent Oxidoreductase,nFAS,OA-519,SDR27X1	fatty acid synthase	Cytoplasm	enzyme
66	FIP1L1	B4DTW7	1300019H17RIK,1300019h17Riken,DKFZp586K0717,factor interacting with PAPOLA and CPSF1,FIP1,hFip1,Rhe,Rje	factor interacting with PAPOLA and CPSF1	Nucleus	other
67	FLNB	O75369	ABP-278,ABP-280,AL024016,AOI,Beta-filamin,FH1,Filamin 3,Filamin B,Filamin homolog 1,filamin, beta,filamin, β,FLN1L,FLN3,Flnb predicted,LRS1,SCT,TABP,TAP,Thyroid autoantigen,β-filamin	filamin B	Cytoplasm	other
68	GNAI3	P08754	87U6,AI158965,ARCND1,AW537698,G alpha I3,G protein subunit alpha i3,G protein subunit α i3,G α I3,G-I3,G-protein α i3,GI alpha3,GIP3A,guanine nucleotide binding protein (G protein), alpha inhibiting 3,guanine nucleotide binding protein (G protein), α inhibiting 3,STIMULATORY G	G protein subunit alpha i3	Cytoplasm	enzyme
69	GNL2	Q5T0F3	BC003262,G protein nucleolar 2,guanine nucleotide binding protein-like 2 (nucleolar),HUMAUANTIG,LOC633966,NGP1,Nog2,Nug2,RGD1305006	G protein nucleolar 2	Nucleus	enzyme
70	GNL3L	Q05DU1	BC020354,G protein nucleolar 3 like,GNL3B,guanine nucleotide	G protein nucleolar	Nucleus	other

			binding protein-like 3 (nucleolar)-like	3 like		
71	GTF3C4	Q05CN7	5330400C03,AI426938,AU014771,AU017413,general transcription factor IIIC subunit 4,general transcription factor IIIC, polypeptide 4,KAT12,LOC687576,TF3C-delta,TF3C-δ,TFIII90,TFIIIC290,TFIIIC90,TFIIICDELTA	general transcription factor IIIC subunit 4	Nucleus	transcription regulator
72	GTF3C5	Q5T7U1	2700084A09RIK,general transcription factor IIIC subunit 5,general transcription factor IIIC, polypeptide 5,HLH Transcription Factor 4,TFiiiC2-63,TFIIIC63,TFIIICepsilon	general transcription factor IIIC subunit 5	Nucleus	transcription regulator
73	GTPBP6	O43824	AV119224,GTP binding protein 6 (putative),LOC100132347,LOC100133897,LOC100292716,LOC100508214,LOC100509054,LOC100510550,LOC100510565,Pgbpll,PGPL	GTP binding protein 6 (putative)	Other	other
74	HCFC1	P51610	AW060991,CFF,HCF,HCF-1,HFC1,host cell factor C1,MRX3,PPP1R89,RGD1563804,VCAF	host cell factor C1	Nucleus	transcription regulator
75	HIST1H1C	P16403	0610008C09Rik,H1.2,H1C,H1F2,H1s-1,H1var1,His1a,histone cluster 1 H1 family member c,histone cluster 1, H1c,Histone h1, member 2,Histone h1.2	histone cluster 1 H1 family member c	Nucleus	other
76	HKDC1	B4E0J9	BC016235,FLJ22761,hexokinase domain containing 1	hexokinase domain containing 1	Cytoplasm	kinase
77	HNRNPD	H0YA96	AUF1,AUF1A,C230004L04,heterogeneous nuclear ribonucleoprotein D,HnRNP UP2,hnRNPD0,HNRPD,P37	heterogeneous nuclear ribonucleoprotein D	Nucleus	transcription regulator
78	HNRNPDL	O14979	AA407431,AA959857,D5Ertd650e,D5Wsu145e,heterogeneous nuclear ribonucleoprotein D-like,HNRDL,HNRNP,HNRNP-D LIKE,Hnrnpdl1,HNRPDL,JKTBP,JKTBP1,JKTBP2,laAUF1,LGMD1G	heterogeneous nuclear ribonucleoprotein D like	Nucleus	other
79	HP1BP3	B0QZK4	heterochromatin protein 1 binding	heterochr	Nucleus	other

			protein 3,heterochromatin protein 1, binding protein 3,HP1-BP74	omatin protein 1 binding protein 3		
80	HSP90AA1	K9JA46	AL024080,AL024147,EL52,Heat shock protein 1, alpha,Heat shock protein 1, α,heat shock protein 90 alpha family class A member 1,heat shock protein 90 α family class A member 1,heat shock protein 90, alpha (cytosolic), class A member 1,heat shock protein 90, α (cytosolic), class A member 1,HEL-S-65p,Hsp103,hsp4,HSP86,Hsp86-1,Hsp89,Hsp89-alpha-delta-N,Hsp89-α-δ-N,HSP89A,Hsp90,HSP90-alpha,HSP90-α,HSP90A,HSP90N,HSPC1,HSPCA, HSPCAL1,HSPCAL4,HSPN,LAP-2	heat shock protein 90 alpha family class A member 1	Cytoplasm	enzyme
81	IGHMBP2	F5H5K3	AEP,CATF1,CMT2S,HCSA,HMN6,immunoglobulin mu binding protein 2,immunoglobulin mu DNA binding protein 2,nmd,RIPE3b1,sma,SMARD1,Smbp-2, SMUBP2,ZFAND7	immunoglobulin mu DNA binding protein 2	Nucleus	enzyme
82	IGKV2-29	A2NJV5	A18,A18b,IGKV,IGVK-A18,immunoglobulin kappa variable 2-29 (gene/pseudogene),immunoglobulin κ variable 2-29 (gene/pseudogene)	immunoglobulin kappa variable 2-29 (gene/pseudogene)	Other	other
83	ITPR3	Q14573	inositol 1,4,5-triphosphate receptor 3,inositol 1,4,5-trisphosphate receptor type 3,inositol 1,4,5-trisphosphate receptor, type 3,IP3R,IP3R-3,IP3R3X,tf	inositol 1,4,5-trisphosphate receptor type 3	Cytoplasm	ion channel
84	KIF23	Q02241	3110001D19Rik,C87313,CHO1,kinesin family member 23,Kinesin-like 5,KNSL5,MKLP-1	kinesin family member 23	Cytoplasm	other
85	KIF2A	B0AZS5	C530030B14RIK,CDCBM3,HK2,KHC2,KIF2,Kinesin 2,kinesin family member 2A,KNS2,M-kinesin	kinesin family member 2A	Cytoplasm	other

86	KIFC1	B4DMA8	Gm4137,HSET,KIFC5A,Kifc5b,kinesin family member C1,kinesin family member C5B,KINESIN-RELATED,KNSL2,Knsl2a,LOC100044006,LOC100044746,MHCB,MKLP2,Tctex-7,Tctex-7A	kinesin family member C1	Nucleus	enzyme
87	KRT13	K7ERE3	CK13,K13,Ka13,Ker47,keratin 13,Keratin complex 1, acidic, gene 13,Krt-1.13,KRT1-13,WSN2	keratin 13	Cytoplasm	other
88	KRT18	B2RA03	48-kD Keratin,CkD,CK-18,CYK18,K18,K1cr,keratin 18,Keratin complex 1 acidic gene 18,KRT1-18	keratin 18	Cytoplasm	other
89	KRT19	P08727	AI663979,CK19,EndoC,K19,K1CS,Ka19, keratin 19,Krt-1.19,Krt1-19	keratin 19	Cytoplasm	other
90	KRT2	P35908	BB005427,CK-2e,K2e,keratin 2,Krt2-2,KRT2A,KRT2E,KRTE	keratin 2	Cytoplasm	other
91	KRT20	P35900	9030623C06RIK,CD20,CK-20,CYTOKERATIN20,K20,KER20,keratin 20,KRT21	keratin 20	Cytoplasm	other
92	KRT78	Q8N1N4	2310030B04Rik,CK-78,K5B,K78,Kb40,keratin 78	keratin 78	Cytoplasm	other
93	KRT8	P05787	AA960620,AL022697,AU019895,CARD2,Ck A,CK-8,CYK8,CYKER,Ecp,EndoA,K2C8,K8,Ker8,keratin 8,Keratin complex 2, basic, gene 8,KO,Krt-2.8,Krt2-8	keratin 8	Cytoplasm	other
94	LGALS3BP	B4DVE1	90K,BTBD17B,CyCAP,Gal-3BP,galectin 3 binding protein,gp90,lectin, galactoside-binding, soluble, 3 binding protein,M2BP,MAC-2-BP,mama,PPICAP,TANGO10B	galectin 3 binding protein	Plasma Membrane	transmembrane receptor
95	LMNA	Q5TCI8	CDCD1,CDDC,CMD1A,CMT2B1,Dhe,EMD2,FPL,FPLD,FPLD2,HGPS,IDC,lamin A,LAMIN A/C,LAMININ A/C,LDP1,LFP,LGMD1B,LMN1,LMNC,LMNL1,MADA,PRO1	lamin A/C	Nucleus	other
96	LMO7	E9PMP7	C130003G01,C78582,E030010D13,FBX20,FBXO20,Gm914,KIAA0858,LIM domain 7,LIM domain only 7,LMO7b,LOMP,mKIAA0858,ZFDCP	LIM domain 7	Cytoplasm	enzyme
97	LONP1	K7EJE8	1200017E13Rik,CODASS,hLON,LON,lon peptidase 1, mitochondrial,LonHS,LONP,Mitochondrial LON Protease,PIM1,Protease,	lon peptidase 1, mitochon	Cytoplasm	peptidase

			serine, 15,PRSS15	drial		
98	LRWD1	H7C5S6	1200011O22Rik,AU042569,AW548074,CENP-33,leucine-rich repeats and WD repeat domain containing 1,ORCA	leucine rich repeats and WD repeat domain containing 1	Nucleus	other
99	LSG1	Q9H089	5830465I20,AA409273,D16Bwg1547e, large 60S subunit nuclear export GTPase 1,RGD1309089	large 60S subunit nuclear export GTPase 1	Cytoplasm	enzyme
100	LUC7L3	Q6PKC2	3300001P08Rik,CISPLATIN RESISTANCE,CRA,CREAP-1,CROP,hLuc7A,LUC7-like 3 (S. cerevisiae),LUC7-like 3 pre-mRNA splicing factor,LUC7A,OA48-18,RGD1307981	LUC7 like 3 pre-mRNA splicing factor	Nucleus	other
101	LYAR	A8K3Y5	Ly1 antibody reactive,Ly1 antibody reactive clone,MLZ-264,ZC2HC2,ZLYAR	Ly1 antibody reactive	Plasma Membrane	other
102	MARS	A8K492	CMT2U,ILFS2,ILLD,methionine-tRNA synthetase,methionyl-tRNA synthetase,METRS,MRS,MTRNS,SPG70	methionyl-tRNA synthetase	Cytoplasm	enzyme
103	MRPS31	Q92665	1500002D03Rik,AA960481,Imogen38, Imogen44,IMOGN38,mitochondrial ribosomal protein S31,S31mt	mitochondrial ribosomal protein S31	Cytoplasm	other
104	MYH10	P35580	5730504C04Rik,9330167F11Rik,Fltn,HSME6,IIB,MCH-B,MHC IIB,MIIB,mKIAA3005,MYHN2,myosin heavy chain 10,Myosin IIB,myosin, heavy polypeptide 10, non-muscle,Nm HC myosin B,NM Myosin-HC,NMHC II-B,NMHC-B,NMMHC-B,NMMHC-IIB, Nonmuscle myosin II,NONMUSCLE MYOSIN2B,SMemb,SMemb variant	myosin heavy chain 10	Cytoplasm	enzyme
105	MYH14	Q7Z406	2400004E04RIK,DFNA4,DFNA4A,FP17425,II-C,Kv3,MHC IIC,MHC16,MYH17,myosin,myosin	myosin heavy chain 14	Extracellular Space	enzyme

			heavy chain 14,Myosin IIC,myosin, heavy polypeptide 14,NHMCII,NMHC II,NMHC-II-C,PNMHH			
106	MYH9	P35579	BDPLT6,DFNA17,EPSTS,FLTN,FTNS,MATINS,MHA,MIIA,MNMM IIA,Myhn-1,Myosib IIA,MYOSIN HEAVY CHAIN 9,Myosin IIA,myosin, heavy chain 9, non-muscle,myosin, heavy polypeptide 9, non-muscle,NM-IIA,NMHC-II-A,NMMHC-A,NMMHC-IIA,NONMUSCLE MYOSIN2 HEAVY CHAIN type A,TU72.6	myosin heavy chain 9	Cytoplasm	enzyme
107	MYO1D	O94832	9930104H07RIK,AW544947,D11Ertd9e,LOC102724697,Myo1c,myosin ID,myosin-1d,Myr2,Myr4,PPP1R108	myosin ID	Cytoplasm	enzyme
108	MYO5B	Q7Z7A5	AI661750,LOC269036,mKIAA1119,Myosin 5b,Myosin VB,Myr6	myosin VB	Cytoplasm	enzyme
109	NARS2	H0YEL9	AI875199,asnRS,asparaginyl-tRNA synthetase 2 (mitochondrial)(putative),asparaginyl-tRNA synthetase 2, mitochondrial,DFNB94,RGD1305145,SLM5	asparaginyl-tRNA synthetase 2, mitochondrial	Cytoplasm	enzyme
110	NCBP1	Q09161	AU014645,AW538051,CBP80,NCBP,NCBP 80 kDa subunit,Nuclear cap binding protein 80 kd,nuclear cap binding protein subunit 1,Sto1	nuclear cap binding protein subunit 1	Nucleus	other
111	NGDN	Q8NEJ9	1500001L15Rik,C14orf120,CANu1,LCP5,lpd-2,neuroguidin,neuroguidin, EIF4E binding protein,NGD,RGD1310211	neuroguidin	Nucleus	other
112	NMNAT1	Q53GL1	2610529L11Rik,5730441G13Rik,D4Cole1e,LCA9,nicotinamide nucleotide adenylyltransferase 1,NMNAT,PNAT1	nicotinamide nucleotide adenylyltransferase 1	Nucleus	enzyme
113	NOC2L	Q9H9J5	AA410003,AF155546,Gm3811,Inhibitor of histone deacetyltransferase,NET15,NET7,NIR, NOC2-like nucleolar associated transcriptional repressor,PPP1R112,RGD1309387	NOC2 like nucleolar associated transcriptional repressor	Nucleus	transcription regulator

114	NUMA1	Q14980	41/7 NMP,6720401E04Rik,AA764025,AL022610,AU014979,NMP 41/7,NMP-22,NUCLEAR MATRIX ANTIGEN,nuclear mitotic apparatus protein 1,NUMA	nuclear mitotic apparatus protein 1	Nucleus	other
115	NUP205	Q92621	3830404O05Rik,AV248391,C7ORF14,KIAA0225,mKIAA0225,NPHS13,nucleoporin 205,Nucleoporin Nup205,Riken cDNA 3830404o05,Similar to CG11943 gene product	nucleoporin 205	Nucleus	other
116	PBRM1	H0Y5B5	2610016F04Rik,AI507524,BAF180,PB1,polybromo 1,RGD1565549	polybromo 1	Nucleus	other
117	PCNA	Q6FI35	ATLD2,Pcna/cyclin,PCNAR,proliferating cell nuclear antigen	proliferating cell nuclear antigen	Nucleus	enzyme
118	PDCD11	Q14690	1110021I22Rik,ALG-4,KIAA0185,mKIAA0185,NFBP,Pdcd7,programmed cell death 11,RRP5	programmed cell death 11	Nucleus	other
119	PDIA6	Q15084	1700015E05Rik,AL023058,C77895,CaBP1,CaBP5,ERP5,LOC100046302,P5,Pdi-p5,PDIA6 isoform 2,PDIR5,Protein disulfide isomerase A6 precursor,protein disulfide isomerase associated 6,protein disulfide isomerase family A member 6,protein disulfide isomerase family A, member 6,Similar to disullide isomerase related,TXNDC7	protein disulfide isomerase family A member 6	Cytoplasm	enzyme
120	PLEC	Q15149	AA591047,AU042537,EBS1,EBSMD,EBSND,EBSO,EBSOG,EBSPA,HD1,LGMD2Q,PCN,PLEC1,PLEC1b,Plectin,PLTN	plectin	Cytoplasm	other
121	PNO1	F8WBJ6	1810003N24RIK,AA407421,AU015468,AU019495,AU042323,Emi3,Imi3,KHRBP1,LOC56902,partner of NOB1 homolog,RRP20	partner of NOB1 homolog	Nucleus	other
122	POLR1C	H0Y723	AA409007,AA959927,Ac2-127,AC40,AL024089,HLD11,Polr1e,polymerase (RNA) I polypeptide C,RNA polymerase I and III subunit C,RPA39,RPA40,RPA5,RPAC1,RPC40,RPO1-1,TCS3	RNA polymerase I and III subunit C	Nucleus	enzyme

123	POLR2A	P24928	hRPB220,hsRPB1,Pol II Largest Subunit,Pol II ls,POLR2,POLRA,polymerase (RNA) II,polymerase (RNA) II (DNA directed) polypeptide A,RNA pol iia,RNA POLYMERASE II LARGE subunit,RNA polymerase II subunit A,RNAP II,RNAP LS,RNAPIIA,RPB1,RPBh1,Rpii215,RpIIL S,RPO2,Rpo2-1,RPOL2	RNA polymerase II subunit A	Nucleus	enzyme
124	PPIB	P23284	AA408962,AA553318,AI844835,Cphn-2,CyP-20b,CYP-S1,CYPB,HEL-S-39,OI9, peptidylprolyl isomerase B,SCYLP	peptidylprolyl isomerase B	Cytoplasm	enzyme
125	PRKDC	P78527	AI326420,AU019811,DNA-DEPENDENT protein KINASE,DNA-PKC,DNA-PKcs,DNAPDcs, DNAPK,DNPK1,DOXNPH,dxnph,HYRC, HYRC1,IMD26,p350,p460,Prkdc predicted,protein kinase, DNA activated, catalytic polypeptide,protein kinase, DNA-activated, catalytic subunit,scid,slip,XRCC7	protein kinase, DNA-activated, catalytic subunit	Nucleus	kinase
126	PRPF6	O94906	1190003A07Rik,2610031L17Rik,ANT-1 ,C20orf14,hPrp6,pre-mRNA processing factor 6,pre-mRNA splicing factor 6,PRP6,PUTATIVE MITOCHONDRIAL OUTER MEMBRANE protein IMPORT receptor,RGD1307103,RP60,SNRNP102,TOM,U5 snRNP-associated 102 kDa,U5-102K	pre-mRNA processing factor 6	Nucleus	transcription regulator
127	PRRC2C	Q9Y520	1810043M20RIK,9630039I18Rik,A630006J20,BAT2-iso,Bat2d,BAT2D1,BAT2L2,E130112L15Rik,mKIAA1096,proline-rich coiled-coil 2C,Prrc3,XTP2	proline rich coiled-coil 2C	Cytoplasm	other
128	PSMA4	H0YLS6	20S PROTEASOME alpha 4 subunit,20S PROTEASOME α 4 subunit,C9,HC9,HsT17706,Macropain subunit C9,proteasome (prosome, macropain) subunit, alpha type 4,proteasome (prosome, macropain) subunit, α type 4,proteasome subunit alpha 4,Proteasome subunit	proteasome subunit alpha 4	Cytoplasm	peptidase

			c9,proteasome subunit α 4,Proteosome subunit α 4,PSC9			
129	PURB	Q96QR8	2310015K15Rik,AA114818,Cager-2,D11BWG0414E,LOC100503489,Pur β,PURBETA,purine rich element binding protein B	purine rich element binding protein B	Nucleus	transcription regulator
130	QARS	Q53HS0	1110018N24Rik,1200016L19Rik,C80286,GLNRS,glutamine--tRNA ligase-like,GLUTAMINYL TRANSSYNTHASE,glutaminyl-tRNA synthetase,LOC100911132,MSCCA,PRO2195	glutaminyl-tRNA synthetase	Cytoplasm	enzyme
131	RALY	Q5QPL9	AI663842,hnRNP-associated with lethal yellow,HNRPCL2,Merc,P542,RALY heterogeneous nuclear ribonucleoprotein,RNA binding protein Raly/Merc	RALY heterogeneous nuclear ribonucleoprotein	Nucleus	transcription regulator
132	RBM25	P49756	fSAP94,NET52,RED120,RNA binding motif protein 25,RNPC7,S164,Snu71,U1 small ribonucleoprotein 1	RNA binding motif protein 25	Nucleus	other
133	RBM47	D6R9D6	9530077J19Rik,FLJ20273,NET18,RGD1359713,RNA binding motif protein 47	RNA binding motif protein 47	Nucleus	other
134	RCN2	H0YL43	AA408742,E6BP,ERC-55,Reticulocalbin 2,TCBP49	reticulocalbin 2	Cytoplasm	other
135	RNF2	Q99496	AI326319,AI450156,AU019207,BAP-1,DING,HIPI3,ring finger protein 2,RING1B,RING2	ring finger protein 2	Nucleus	transcription regulator
136	RNMT	K7EP06	2610002P10Rik,AI848273,cm1p,CMT1,CMT1c,hCMT1,hMet,MET,mKIAA0398,RG7MT1,RNA (guanine-7-) methyltransferase,RNA guanine-7 methyltransferase	RNA guanine-7 methyltransferase	Nucleus	enzyme
137	RPL13	Q6NZ55	A52,BBC1,D16S444E,D16S44E,Gm10071,L13,ribosomal protein L13,ribosomal protein L13, pseudogene 6,Rpl13-ps6	ribosomal protein L13	Nucleus	other
138	RPL17	J3QS96	ASI,Gm14934,L17,LOC100133240,PD-1,ribosomal protein L17,ribosomal	ribosomal protein	Cytoplasm	other

			protein L17, pseudogene 8,Rpl17-ps8,RPL23	L17		
139	RPL21	Q6IAX2	8430440E03Rik,HYPT12,L21,ribosomal protein L21	ribosomal protein L21	Cytoplasm	other
140	RPL7L1	Q6DKI1	1500016H10Rik,dJ475N16.4,RGD1308469,ribosomal protein L7-like 1	ribosomal protein L7 like 1	Cytoplasm	transcription regulator
141	RPN2	P04844	1300012C06Rik,AV261018,RIBIIR,ribophorin II,RPN-II,SWP1	ribophorin II	Cytoplasm	enzyme
142	RPP30	Q5VU11	LOC283012,LOC687772,ribonuclease P/MRP 30 subunit,ribonuclease P/MRP subunit p30,RNASEP2,TSG15	ribonuclease P/MRP subunit p30	Nucleus	enzyme
143	RPS6	Q96DV6	40S ribosomal protein S6-like,LOC100911372,pp33,Q9BZU1, RIBOSOMAL protein S6,S6,S6R,S6RP	ribosomal protein S6	Cytoplasm	other
144	RUVBL2	Q9Y230	CGI-46,ECP-51,INO80J,mp47,p47,REPTIN,RuvB like DNA helicase,RuvB-like AAA ATPase 2,RuvB-like protein 2,RUVB2,RVB2,TAP54-beta,TAP54-β,TIH2,TIP48,TIP49B	RuvB like AAA ATPase 2	Nucleus	transcription regulator
145	SAP30BP	Q9UHR5	2700016D05Rik,HCNGP,HTRG,HTRP,RGD1565537,SAP30 binding protein	SAP30 binding protein	Nucleus	transcription regulator
146	SART1	B4DDH9	Ara1,HAF,HOMS1,SART1, U4/U6.U5 tri-snRNP-associated protein 1,SART1259,SNRNP110,Snu66,SPF107, squamous cell carcinoma antigen recognized by T cells 1,SSCA1,U5-110K	SART1, U4/U6.U5 tri-snRNP-associated protein 1	Nucleus	other
147	SF3B4	Q53FG6	AFD1,Hsh49,SAP49,SF3b49,SF3b50,SPLICING FACTOR 3B subunit4,splicing factor 3b, subunit 4	splicing factor 3b subunit 4	Nucleus	other
148	SFPQ	Q9BSV4	1110004P21RIK,2810416M14Rik,5730453G22RIK,9030402K04Rik,AU021830,D4Ertd314e,Gm12940,LOC100504686,LOC100506168,LOC252855,Myoblast Antigen 24.1D5,POMP100,PPP1R140,PSF,Ptb associated splicing factor,REP1,splicing factor proline and glutamine rich,splicing factor proline/glutamine	splicing factor proline and glutamine rich	Nucleus	transcription regulator

			rich (polypyrimidine tract binding protein associated)			
149	SFXN3	Q9BWM7	BA108L7.2,LOC65042,SFX3,sideroflexin 3,SLC56A3,SLC64A3,TCC	sideroflexin 3	Cytoplasm	transporter
150	SIX1	Q15475	BB138287,BOS3,DFNA23,sine oculis-related homeobox 1,SIX homeobox 1,TIP39	SIX homeobox 1	Nucleus	transcription regulator
151	SLC12A2	P55011	9330166H04Rik,BSC,BSC2,mBSC2,Na-k-cl cotransporter,NKCC1,PPP1R141,solute carrier family 12 member 2,solute carrier family 12, member 2,sy-ns	solute carrier family 12 member 2	Plasma Membrane	transporter
152	SLC25A22	E9PJH7	1300006L01RIK,AI060884,EIEE3,GC-1, LOC100911440,mitochondrial glutamate carrier 1-like,NET44,RGD1307826,solute carrier family 25 (mitochondrial carrier, glutamate), member 22,solute carrier family 25 member 22	solute carrier family 25 member 22	Cytoplasm	transporter
153	SLC3A2	P08195	4F2,4F2HC,4T2HC,AI314110,CD98,CD98HC,FRP1,Ly-10,Ly-m10,MDU1,Mgp-2hc,NACAE,solute carrier family 3 (activators of dibasic and neutral amino acid transport), member 2,solute carrier family 3 member 2	solute carrier family 3 member 2	Plasma Membrane	transporter
154	SMAD3	H0YMP2	AU022421,DKFZP586N0721,hMAD-3, HSPC193,HsT17436,JV15-2,LDS1C,LDS3,MAD3,MADH3,SMAD family member 3	SMAD family member 3	Nucleus	transcription regulator
155	SMARCA4	P51532	b2b508.1Clo,b2b692Clo,BAF190,BAF190A,BRG,BRG1,CSS4,HP1-BP72,hSNF2b,MRD16,RTPS2,SNF2,SNF2-beta,SNF2-β,SNF2L4,SNF2LB,SW1/SNF,SWI/SNF related, matrix associated, actin dependent regulator of chromatin, subfamily a, member 4,SWI2	SWI/SNF related, matrix associated, actin dependent regulator of chromatin, subfamily a, member 4	Nucleus	transcription regulator

156	SMCHD1	A6NHR9	4931400A14Rik,AW554188,BAMS,ENSMUSG00000041913,FSHD2,mKIAA0650,MommeD1,RGD1307234,SMC hinge domain containing 1,structural maintenance of chromosomes flexible hinge domain containing 1	structural maintenance of chromosomes flexible hinge domain containing 1	Nucleus	enzyme
157	SNRNP70	P08621	2700022N21Rik,3200002N22Rik,AI325098,R74807,RNPU1Z,Rnu1p70,Rnulp70,RPU1,small nuclear ribonucleoprotein 70 (U1),small nuclear ribonucleoprotein U1 subunit 70,Snp1,SNRP70,Srnp70,U1 SNRNP 70K,U1 SNRNP70,U1-70,U1-70K,U1AP,U1RNP	small nuclear ribonucleoprotein U1 subunit 70	Nucleus	other
158	SNRPA	M0R268	C430021M15RIK,Mud1,Rnu1a-1,small nuclear ribonucleoprotein polypeptide A,U1 SNRNP-SPECIFIC protein A,U1-A	small nuclear ribonucleoprotein polypeptide A	Nucleus	other
159	SNRPD1	J3QLI9	AA407109,AL023031,HsT2456,small nuclear ribonucleoprotein D1,small nuclear ribonucleoprotein D1 polypeptide,SMD1,Smn,snRNP D1,SNRPD	small nuclear ribonucleoprotein D1 polypeptide	Nucleus	other
160	SON	P18583	2900011L12Rik,AA409051,AU067731,BASS1,C21orf50,C81487,DBP-5,DNA-5B,mKIAA1019,NREBP,SON DNA binding protein,SON3,TOKIMS	SON DNA binding protein	Nucleus	other
161	SPOUT1	Q5T280	9930028P05,AL033358,C9orf114,CENP-32,D2Wsu81e,HSPC109,SPOUT domain containing methyltransferase 1	SPOUT domain containing methyltransferase 1	Nucleus	other
162	SPTAN1	Q13813	(alpha)II-SPECTRIN,(α)II-SPECTRIN,2610027H02Rik,A2a,Alpha fodrin,Alpha spectrin,alpha SPECTRIN 2,Alpha-spectorin,AlphaII spectrin,EIEE5,Fodrin,IPF,NEAS,Spectri	spectrin alpha, non-erythrocytic 1	Plasma Membrane	other

			n alpha 2,spectrin alpha, non-erythrocytic 1,Spectrin α 2,spectrin α, non-erythrocytic 1,spectrin, alpha, non-erythrocytic 1,spectrin, α, non-erythrocytic 1,SPNA2,SPTA2,α fodrin,α spectrin,α SPECTRIN 2,α-spectorin			
163	SPTBN1	B2ZZ89	9930031C03Rik,AL033301,beta II SPECTRIN,beta-Fodrin,betaSpII,ELF,elf 1,ELF3,HEL102,mKIAA4049,spectrin beta, non-erythrocytic 1,SPECTRIN beta1,Spectrin Beta2,spectrin β, non-erythrocytic 1,spectrin, beta, non-erythrocytic 1,spectrin, β, non-erythrocytic 1,Spnb1,SPNB2,SPTB2,β II SPECTRIN,β-Fodrin	spectrin beta, non-erythrocytic 1	Plasma Membrane	other
164	SREK1	B3KRJ9	8430401B01,AI450757,AI462342,AL118220,SFRS12,splicing regulatory glutamic acid and lysine rich protein 1,splicing regulatory glutamine/lysine-rich protein 1,SRrp508,SRrp86,Srsf12	splicing regulatory glutamic acid and lysine rich protein 1	Nucleus	other
165	SRRM2	Q9UQ35	5033413A03Rik,AA410130,Cwc21,CWF21,HSPC075,KIAA0324,mKIAA0324,serine/arginine repetitive matrix 2,SRL300,SRm300	serine/arginine repetitive matrix 2	Nucleus	other
166	SRRT	Q9BXP5	2810019G02RIK,ARS2,ASR2,ASR2A,ASR2B,ASR2C,ASR2D,serrate,serrate RNA effector molecule homolog (Arabidopsis),serrate, RNA effector molecule	serrate, RNA effector molecule	Nucleus	other
167	SRSF10	O75494	FUSIP1,FUSIP2,LOC100505793,LOC100996657,LOC642558,LOC727922,NSSR,NSSR1,NSSR2,PPP1R149,serine and arginine rich splicing factor 10,serine/arginine-rich splicing factor 10,SFRS13,SFRS13A,SRp38,SRrp40,Srsf13a,TASR,TASR1,TASR2,TLS-associated serine-arginine	serine and arginine rich splicing factor 10	Nucleus	other
168	SRSF5	Q13243	HRS,serine and arginine rich splicing factor 5,SFRS5,SRP40	serine and arginine rich splicing	Nucleus	other

				factor 5		
169	SRSF7	Q16629	9430065L19Rik,9G8,AAG3,NX-96,serine and arginine rich splicing factor 7,serine/arginine-rich splicing factor 7,SFRS7	serine and arginine rich splicing factor 7	Nucleus	other
170	SUPT6H	Q7KZ85	5131400N11RIK,AI132449,emb-5,mKIAA0162,SPT6,SPT6 homolog, histone chaperone,SPT6H,SPTH6,suppressor of Ty 6,Supt6	SPT6 homolog, histone chaperone	Nucleus	transcription regulator
171	TBL2	B4DY59	C76179,transducin (beta)-like 2,transducin (β)-like 2,transducin beta like 2,transducin β like 2,WBSCR13,WS-betaTRP,WS-bTRP	transducin beta like 2	Plasma Membrane	other
172	TBL3	A0JLS5	9430070M15Rik,SAZD,transducin (beta)-like 3,transducin (β)-like 3,transducin beta like 3,transducin β like 3,UTP13	transducin beta like 3	Cytoplasm	peptidase
173	TEFM	Q96QE5	1110002N22Rik,C17orf42,RGD1306595,transcription elongation factor, mitochondrial	transcription elongation factor, mitochondrial	Cytoplasm	transcription regulator
174	TFB2M	Q9H5Q4	Hkp1,mtTFB2,transcription factor B2, mitochondrial	transcription factor B2, mitochondrial	Cytoplasm	enzyme
175	THAP11	Q96EK4	2810036E22RIK,AB041579,CTG-B43a, CTG-B45d,HRIHFB2206,RONIN,THAP domain containing 11	THAP domain containing 11	Nucleus	transcription regulator
176	THOC2	Q8NI27	6330441O12RIK,CXorf3,D130005M13, D130005M13Rik,dJ506G2.1,Gm1139, Gm1793,hTREX120,MRX12,MRX35,RGD1561623,THO complex 2,THO2	THO complex 2	Nucleus	other
177	TJP1	Q07157	Gm1693,tight junction protein 1,ZO-1,zona occludens 1	tight junction protein 1	Plasma Membrane	other
178	TJP2	Q9UDY2	C9DUPq21.11,DFNA51,DUP9q21.11,PFIC4,tight junction protein 2,X104,ZO-2	tight junction protein 2	Plasma Membrane	kinase

179	TMA16	D6RA57	1810029B16Rik,C4orf43,Hypothetical protein FLJ11184,RGD1305222,translation machinery associated 16,translation machinery associated 16 homolog	translation machinery associated 16 homolog	Nucleus	other
180	TMPO	P42166	5630400D24Rik,AI195756,AI606875,AW214352,AW547477,CMD1T,LAP2,Lap2 alpha,Lap2 α,LEMD4,PRO0868,thymopoietin,TP,TP alpha,TP beta/gamma,TP α,TP β/γ	thymopoietin	Nucleus	other
181	TOE1	B4DP23	4930584N22RIK,4933424D16Rik,AI413517,CAF1Z,hCaf1z,PCH7,target of EGR1, exonuclease,target of EGR1, member 1 (nuclear)	target of EGR1, exonuclease	Nucleus	enzyme
182	TOP2A	P11388	DNA TOPOISOMERASE II alpha,DNA TOPOISOMERASE II α,TOP2,Top2a: topoisomerase (dna) ii α (170kd),TOPO2 alpha,TOPO2 α,topoisomerase (DNA) II alpha,topoisomerase (DNA) II α,Topoisomerase II alpha,Topoisomerase II α,TP2A	DNA topoisomerase II alpha	Nucleus	enzyme
183	TOP2B	E9PCY5	D230016L12RIK,DNA topoisomerase II beta,DNA topoisomerase II β,Top-2,top2beta,TOPIIB,Topo2 β,topoisomerase (DNA) II beta,topoisomerase (DNA) II β,Topoisomerase ii β	DNA topoisomerase II beta	Nucleus	enzyme
184	TP53	P04637	bbl,BCC7,bfy,bhy,LFS1,p44,P53,P53 cellular tumour antigen,p53 tumor suppressor,transformation related protein 53,TRP53,tumor protein p53,tumour protein p53	tumor protein p53	Nucleus	transcription regulator
185	TPM3	B2RDE1	CAPM1,CFTD,gamma-TM,HEL-189,HEL-S-82p,hscp30,hTM30nm,hTMnm,LOC286890,NEM1,OK/SW-cl.5,TM-5,TM3,TM30,TM30nm,Tm5NM,Tm5nm1-2,TMnm,Tpm-5,TPM3nu,TPMsk3,TRK,Trop-5,tropomyosin 3,tropomyosin 3, gamma,tropomyosin 3, γ,Tropomyosin gamma,Tropomyosin γ,γ-TM	tropomyosin 3	Cytoplasm	other
186	TPM4	K7ERG3	2610528G24RIK,HEL-S-108,Tpm4.2,Tpm4.2cy,tropomyosin 4	tropomyosin 4	Cytoplasm	other

187	TPR	P12270	2610029M07Rik,C77892,FLJ13049,translocated promoter region, nuclear basket protein	translocated promoter region, nuclear basket protein	Nucleus	other
188	TRAM1	G3XAN4	1810049E02Rik,LOC100130862,PNAS8,TRAM,TRAMP,translocating chain-associating membrane protein 1,translocation associated membrane protein 1	translocation associated membrane protein 1	Cytoplasm	other
189	TRIP12	Q14669	1110036I07RIK,6720416K24RIK,AA410158,GTL6,KIAA0045,MRD49,thyroid hormone receptor interactor 12,ULF	thyroid hormone receptor interactor 12	Cytoplasm	enzyme
190	TTBK2	Q8IWY7	2610507N02RIK,AI326283,B930008N24Rik,KIAA0847,mKIAA0847,SCA11,tau tubulin kinase 2,TTBK,Ttbk1,TTK	tau tubulin kinase 2	Cytoplasm	kinase
191	TUFM	P49411	2300002G02RIK,C76308,C76389,COXPD4,EF-TuMT,EFTU,MTEF-TU,P43,Tu translation elongation factor, mitochondrial	Tu translation elongation factor, mitochondrial	Cytoplasm	translation regulator
192	U2SURP	O15042	2610101N10Rik,AU023006,fSAPa,RGD1307882,SR140,U2 snRNP-associated SURP domain containing	U2 snRNP associated SURP domain containing	Nucleus	other
193	UBB	J3QS39	AL033289,HEL-S-50,RPS27A,UBA52,Ubb2,UBC,UBIQUITIN,UBIQUITIN B	ubiquitin B	Cytoplasm	enzyme
194	UBTF	B4DNQ1	A930005G04RIK,CONDBA,LOC679205,NOR-90,TCFUBF,UBF,UBF-1,upstream binding transcription factor,upstream binding transcription factor, RNA polymerase I	upstream binding transcription factor	Nucleus	transcription regulator
195	UQCRC2	H3BSJ9	1500004O06Rik,AURA11,Core 2,CORE protein 2 of COMPLEX III,Cytochrome b-c1 complex subunit 2,MC3DN5,MGC94368,Mitochondrial	ubiquinol-cytochrome c reductase	Cytoplasm	enzyme

			Core Protein2,QCR2,Rikubiquinol Cytochrome C Reductase Core Protein 2,Ubiquinol Cytochrome C Reductase Core Protein 2 Precursor,ubiquinol-cytochrome c reductase core protein 2,UQCR2	core protein 2		
196	UTP20	O75691	1A6/DRIM,3830408P06Rik,AA617408,DRIM,mDRIM,mDRIN,RGD1560606,UTP20 small subunit processome component,UTP20, small subunit processome component	UTP20, small subunit processome component	Nucleus	other
197	UTP25	A8K0P8	AA408296,AW045375,C1orf107,DEF,DIEXF,digestive organ expansion factor homolog (zebrafish),DJ434O14.5,UTP25, small subunit processor component	UTP25, small subunit processor component	Nucleus	other
198	VCP	P55072	3110001E05,CDC48,IBMPFD,p97,p97/VCP,Ter atpase,TERA,Transitional Endoplasmic Reticulum Atpase,valosin-containing protein,XSG7	valosin containing protein	Cytoplasm	enzyme
199	VDAC2	P45880	mVDAC2,mVDAC6,POR,Vdac6,voltage-dependent anion channel 2	voltage dependent anion channel 2	Cytoplasm	ion channel
200	WDR11	Q659C9	2900055P10Rik,AW489876,BRWD2,DR11,HH14,mKIAA1351,SRI1,WD repeat domain 11,WDR15	WD repeat domain 11	Cytoplasm	other
201	WDR3	Q5TDG3	AW546279,D030020G18Rik,DIP2,UTP12,WD repeat domain 3	WD repeat domain 3	Cytoplasm	other
202	YBX1	Q6PKI6	1700102N10RIK,BP-8,Byb1,C79409,CBF-A,CSDA2,CSDB,DBPB,DBPB-LIKE,EF1A,EFI-A,MDR-NF1,MSY1,mYB-1a,NSEP-1,THYROID Y BOX,VACSSBF1,Y box protein 1,Y-box Binding Protein1,YB-1,YEBP	Y-box binding protein 1	Nucleus	transcription regulator
203	YTHDC2	Q6ZMY0	3010002F02RIK,BC037178,CAHL,hYTHDC2,LOC100365272,mYTHDC2,RGD1563192,YTH domain containing 2	YTH domain containing 2	Cytoplasm	enzyme

204	YWHAZ	D0PNI1	1110013I11RIK,14-3-3 delta,14-3-3 ZETA/delta,14-3-3 δ,14-3-3 ζ/δ,14-3-3-zeta,14-3-3-ζ,14-3-3z,AI596267,AL022924,AU020854,HEL-S-3,HEL-S-93,HEL4,KCIP-1,Protein kinase c inhibitor 1 beta/alpha,Protein kinase c inhibitor 1 β/α,Protein kinase C inhibitor protein 1 zeta/delta,Protein kinase C inhibitor protein 1 ζ/δ,Tyr mono,Tyrosine 3-Monooxygenase ζ Polypeptide,tyrosine 3-monooxygenase/tryptophan 5-monooxygenase activation protein zeta,tyrosine 3-monooxygenase/tryptophan 5-monooxygenase activation protein ζ,tyrosine 3-monooxygenase/tryptophan 5-monooxygenase activation protein, zeta,tyrosine 3-monooxygenase/tryptophan 5-monooxygenase activation protein, zeta polypeptide,tyrosine 3-monooxygenase/tryptophan 5-monooxygenase activation protein, ζ,tyrosine 3-monooxygenase/tryptophan 5-monooxygenase activation protein, ζ polypeptide,YMHAZ,YWHAD	tyrosine 3-monooxygenase/tryptophan 5-monooxygenase activation protein zeta	Cytoplasm	enzyme
205	YY1	H0YJV7	AW488674,DELTA,GADEVS,Histone alpha factor,Histone α factor,INO80S,NF-E1,NMP-1,UCRBP,Yin and yang 1,YIN-YANG-1,YING-YANG-1,YY1 transcription factor,δ	YY1 transcription factor	Nucleus	transcription regulator
206	ZC3H18	B4DZ24	1190001B23Rik,5830416A07Rik,NHN1,zinc finger CCCH-type containing 18	zinc finger CCCH-type containing 18	Nucleus	other
207	ZCCHC8	A8K559	5730565F05Rik,LOC100507874,zinc finger CCHC-type containing 8,zinc finger, CCHC domain containing 8	zinc finger CCHC-type containing 8	Nucleus	other
